# Surface Tension and Neuronal Sorting in Magnetically Engineered Brain‐Like Tissue

**DOI:** 10.1002/advs.202302411

**Published:** 2023-08-06

**Authors:** Jose E. Perez, Audric Jan, Catherine Villard, Claire Wilhelm

**Affiliations:** ^1^ Laboratoire Physico Chimie Curie CNRS UMR168 Institut Curie Sorbonne Université PSL University Paris 75005 France; ^2^ Institut Pierre‐Gilles de Gennes IPGG Technology Platform UMS 3750 CNRS Paris 75005 France; ^3^ Laboratoire Interdisciplinaire des Énergies de Demain Université Paris Cité UMR 8236 CNRS Paris 75013 France

**Keywords:** 3D brain model, bioengineering, glia/neuron organization, magnetic nanoparticles, spheroids, surface tension

## Abstract

Engineered 3D brain‐like models have advanced the understanding of neurological mechanisms and disease, yet their mechanical signature, while fundamental for brain function, remains understudied. The surface tension for instance controls brain development and is a marker of cell‐cell interactions. Here, 3D magnetic brain‐like tissue spheroids composed of intermixed primary glial and neuronal cells at different ratios are engineered. Remarkably, the two cell types self‐assemble into a functional tissue, with the sorting of the neuronal cells toward the periphery of the spheroids, whereas the glial cells constitute the core. The magnetic fingerprint of the spheroids then allows their deformation when placed under a magnetic field gradient, at a force equivalent to a 70 g increased gravity at the spheroid level. The tissue surface tension and elasticity can be directly inferred from the resulting deformation, revealing a transitional dependence on the glia/neuron ratio, with the surface tension of neuronal tissue being much lower. The results suggest an underlying mechanical contribution to the exclusion of the neurons toward the outer spheroid region, and depict the glia/neuron organization as a sophisticated mechanism that should in turn influence tissue development and homeostasis relevant in the neuroengineering field.

## Graphical abstract

Neurons self‐sort toward the periphery of magnetic brain‐like tissue spheroids after intermixing with glia. The magnetically reconstituted spheroids show a transition from low to high surface tension that is dependent on the proportion of glia, which provide most of the tissue cohesive signature.

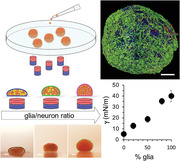

## 1 Introduction

Human brain in vitro 3D models have since recent years been utilized for recapitulating the properties and physiology of this organ with the purpose of studying brain‐penetrating drugs,^[^ ^1^ ^]^ cell‐cell interactions,^[^ ^2^ ^]^ brain disease^[^ ^3^ ^]^ and neurological disorders.^[^ ^4^ ^]^ These models have evolved from 2D organized networks^[^ ^5^ ^]^ to culture in 3D scaffolds or matrices,^[^ ^6^, ^7^, ^8^ ^]^ and more recently to complex organoid systems with defined brain regions that more closely approximate the in vivo brain cell biology.

The response of brain cells to changes in the tissue's mechanical properties is known to play important roles in development, physiology, signaling and pathology.^[^ ^9^ ^]^ It has been evidenced for instance that the stiffness of the cell microenvironment regulates neurite growth^[^ ^10^ ^]^ and extension, as well as self‐renewal and differentiation in neural stem cells.^[^ ^11^ ^]^ Glial cells may also thrive better on softer substrates, provided they are coupled with suitable adhesion coatings.^[^ ^12^ ^]^ The tissue surface tension, a biological marker of intercellular binding energy and hence of tissue cohesivity, has been linked to brain gyrification in embryogenesis.^[^ ^13^ ^]^ The concept of the tissue surface tension arising from the cohesive and adhesive interactions between cell populations and conferring them their liquid‐like behavior was introduced by Steinberg many years ago.^[^ ^14^ ^]^ In brief, it postulates that the liquid‐like behavior of intermixed cell populations closely follows that of immiscible liquids, with cell sorting and arrangement being dependent on the surface tension of each cell population, as well as the interfacial tension between the phases. Such mechanistic analysis of the behavior of different cell populations as they are interfaced with one another is of particular interest in studies of tissue morphogenesis, with implications in the final purpose of recreating functional 3D tissue models.

In humans, the glia/neuron ratio in terms of cell number sits close to 50:50.^[^ ^15^ ^]^ However, how this cell arrangement takes place in the case of neuronal and glial cell populations as they become intermixed, as well as the quantification and contribution of each cell population to the overall tissue mechanical properties, have remained largely unexplored despite their relevancy in in vitro tissue morphogenesis. Here, we hypothesized that different intermixing ratios of glial and neuronal cells would translate to differential tissue mechanical properties. To investigate this idea, we envisioned the engineering of a brain‐like 3D construct that can be physically stimulated in order to retrieve these properties. A rarely met need in 3D tissue engineering is the controlled organization of single cells via remote manipulation. Magnetic forces are probably first‐in‐class candidates for this purpose because they can act at a distance.^[^ ^16^, ^17^, ^18^ ^]^ Cells can become magnetic through the internalization of biocompatible iron oxide nanoparticles, widely used in cancer theragnostics,^[^ ^19^ ^]^ drug delivery,^[^ ^20^ ^]^ and already clinically validated as *T_2_* contrast agents in magnetic resonance imaging,^[^ ^21^ ^]^ with recent advances validating their use for *T_1_* contrast.^[^ ^22^ ^]^ Clinical nanoparticle formulations may additionally exhibit anti‐tumor properties.^[^ ^23^ ^]^

In a self‐integrating, all‐in‐on process, we thus provide here magnetically‐formed 3D tissues shaped in less than a day from individual primary glial and neuronal murine cells labeled with biocompatible iron oxide nanoparticles, and with an inherent capacity for further mechanical stimulation throughout their maturation. These active brain‐like constructs were used to explore the distinct contribution of both glial and neuronal cell populations at different intermixing ratios to tissue surface tension, elasticity and organization.

## 2 Results

### 2.1 Spheroid Formation after Intermixing of Neuronal and Glial Cells

**Figure 1:**
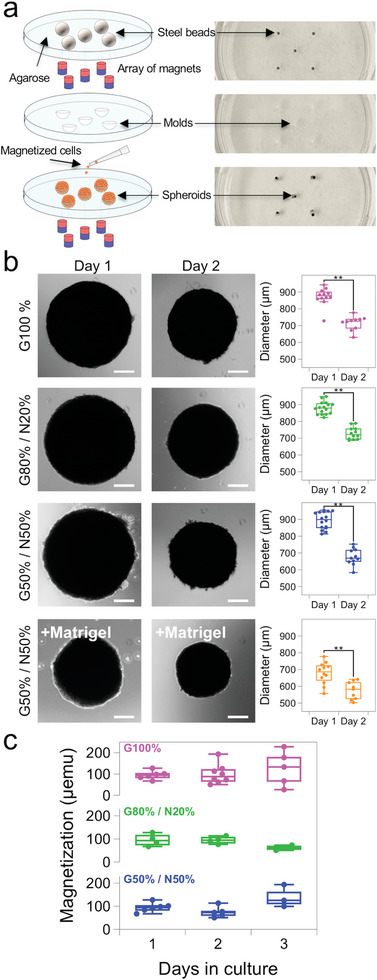
Magnetic molding of neuronal and glial cell spheroids. a) Spherical molds are produced in agarose using a set of steel beads of 1 mm in diameter and held in place with an array of magnets. Upon removal of the beads, cells that have been pre‐labeled with magnetic nanoparticles are attracted into the mold using the same array of magnets in order to obtain spheroids whose shape is defined by the agarose mold. b) Optical images of spheroid formed after intermixing glial and neuronal cell populations at ratios of G100%, G80%/N20%, G50%/N50%, and G50%/N50% pre‐mixed with Matrigel matrix. Box plots show the quantification of spheroid diameter over a period of 2 days, evidencing a significant decrease in size for all conditions. *n* > 3 for all conditions, with each dot corresponding to one measured spheroid; **p < 0.01 (c) Box plots showing the spheroid magnetization quantification over 3 days of culture by vibrating sample magnetometry. *n* ≥ 3 for all conditions, with each dot corresponding to one measured spheroid. Scale bars = 200 µm.

Primary glial and neuronal cells were extracted from brain embryos of mice at embryonic day 15‐16 (see Experimental Section). It is expected that the isolated glial cell population is comprised mostly of astrocytes, with a minimal subset of oligodendrocytes at this embryonic day of extraction.^[^ ^24^, ^25^ ^]^ The presence of neural stem cell progenitors was evidenced in the isolated primary neuronal cells, but not in the glial ones (Figure S1, Supporting Information). We proceeded to use the magnetic molding method to form magnetic spheroids after intermixing of the obtained primary glial and neuronal cells (**Figure** **1**a). It is based on an initial quick labeling and magnetization step of the cells with iron oxide nanoparticles while in suspension (see Experimental Section). Cell viability analysis of primary glial and neuronal cells indicated excellent biocompatibility for the nanoparticle dose and incubation settings (Figure S2 and S3, Supporting Information). First, a series of spherical molds is made by partially submerging steel round beads of 1 mm in diameter in liquid agarose, held in place by an array of magnets below. After gelation of the agarose, the steel beads are removed with the aid of a magnet, leaving behind a perfectly spherical mold. The magnetized cell populations at the desired intermixed ratio are pipetted into the mold, with the magnet array below acting as an attractor and thus compelling the cells to aggregate into a spherical shape as they fill the mold. The methodology results in spheroids with a high level of sphericity and high compaction that translate to maturation times as short as 1 day. More importantly, the methodology can yield magnetic spheroids with an average diameter in the 600–900 µm range, which are more easily deformable under a magnetic field in order to estimate their surface tension (see below).

Five different cell ratios were studied in terms of the percentage of cells composing the spheroid, henceforth denoted as glia/neuron (G/N) ratios: G100%, G80%/N20%, G50%/N50%, G20%/N80%, and N100%. Additional conditions of cells mixed with Matrigel before the spheroid formation process were produced in order to study a possible effect of the presence of an underlying matrix on the mechanical properties of the spheroids. Importantly, a remarkable difference was observed in the expression of E‐cadherin, a marker of cell‐cell adhesion and cohesion, between G100% and N100% spheroids (Figure S4, Supporting Information). This lack of tissue cohesiveness of neurons resulted in fewer viable spheroids produced for the conditions of G20%/N80% and N100%, most of which lost their structural integrity after 1 day of maturation.

Typical spheroids for each of the intermixed cell population conditions are shown in Figure 1b. Remarkably, a consistent spheroid shrinkage and compaction can be observed across all the conditions, as quantified by a decrease in spheroid diameter of 15% on average of the original diameter upon spheroid collection at day 1 of maturation in the mold. Of interest too is the fact that for spheroids with Matrigel intermixed the initial diameter after 1 day of maturation was smaller compared to all the non‐Matrigel conditions. All formed spheroids possess a magnetic fingerprint bestowed by the labeling of each individual cell with the magnetic nanoparticles, and which drives the tissue deformation necessary for the inferring of the mechanical properties proposed in this work. The established cell ratios are expected to be maintained over maturation, considering the non‐proliferative nature of primary neuronal cells, and that the glial cell number does not change significantly over a maturation period of up to 5 days of culture in neurobasal medium (Figure S5, Supporting Information), also previously reported when mixed with neuronal cells.^[^ ^12^ ^]^ Figure 1c shows the evolution of the spheroids’ magnetization over the course of 3 days of maturation, measured by vibrating sample magnetometry at the single spheroid level. The magnetization remains relatively constant over the maturation of the spheroids, in the range of 100 µemu for all conditions, indicating no loss of their magnetic signature over this period of time. We further extended this quantification on G100% spheroids, and found a slight decrease in their magnetic moment after 5 days of culture, albeit in a non‐significant way (Figure S6, Supporting Information).

**Figure 2:**
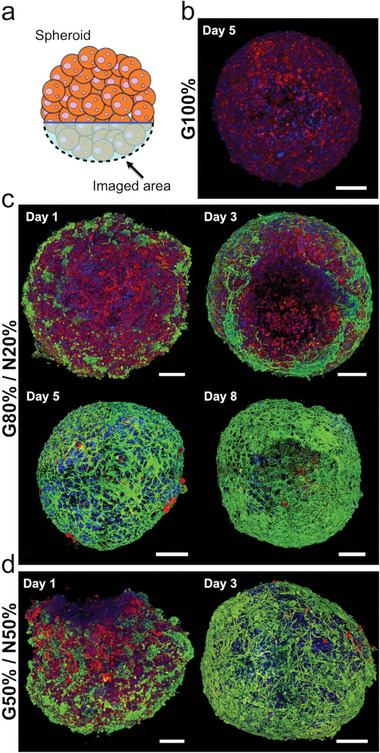
Fluorescence imaging 3D reconstruction of spheroids after glial and neuronal cell intermixing and magnetic molding. a) Diagram showing the imaged area of the spheroids. b) G100% spheroid at day 5 of maturation c) Spheroids at a ratio of G80%/N20% after 1, 3, 5, and 8 days of maturation. d) Spheroids at a ratio of G50%/N50% after 1 and 3 days of maturation. Fluorescence images show β‐tubulin III in green, GFAP in red and DAPI in blue. *n* = 3 for each imaged ratio. Scale bars = 100 µm.

Direct observation of the spheroids through fluorescence microscopy revealed the arrangement architecture of the neuronal and glial cell populations. For this purpose, we performed fluorescence imaging of the whole spheroid, from which we produced a 3D reconstruction of the imaged area that represents a visual depth of field of view of ≈200 µm into the tissue (**Figure** **2**a). In G100% spheroids, the red glial fibrillary acidic protein (GFAP)‐stained glial cells appear to be evenly distributed across the spheroid structure (Figure 2b). Interestingly, with the addition of neuronal cells, the glial cell population appears to remain in the core of the spheroid structure, with neuronal cells arranging themselves toward the periphery. For the G80%/N20% ratio spheroids at day 1 of maturation, neuronal cells cluster in small groups along the periphery of the spheroid, observable by β‐tubulin III staining in green (Figure 2c). Significant elongation of neuronal processes, presumably axons for the longest ones, is seen inside and in‐between these clusters. The development of neuronal branches is more advanced at day 3 of maturation, promoting the connection of initially isolated neuronal clusters. At days 5 and 8 of maturation, the neuronal network density is remarkably increased, shrouding the GFAP red signal. Increasing the neuronal density to 50% induces the presence of even more neuronal clusters observed at day 1 (Figure 2d). After 3 days, neuronal processes have fully spread at the periphery of the spheroid, with multiple axonal branching and networks appearing to completely cloud over the (red‐stained) glial cell population, as in the previous condition. Taken together, these imaging results reveal a possible specific arrangement where the neuronal cells group themselves in the outer shell of the spheroid, establishing cell‐cell communication and axonal networks on top of an inner core of glial cells. We then evaluated whether this structural organization was maintained in non‐magnetic spheroids prepared in agarose wells under centrifugation (Figure S7, Supporting Information). After 2 days of maturation, no apparent organization could be observed, with cells appearing loosely aggregated in a non‐spherical way. At day 5, a more packed spherical structure can be inferred, yet with no evident cell organization.

**Figure 3:**
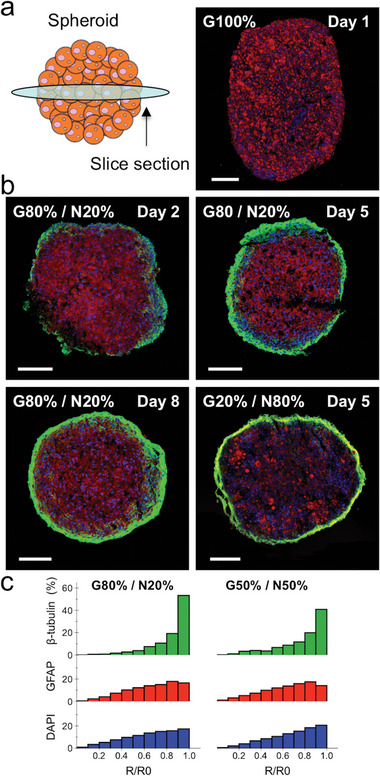
Spheroid cross‐section fluorescence imaging. a) Spheroid cross‐sections were obtained from the center of the spheroid by cryo‐sectioning; cross‐section fluorescence imaging of G100% spheroid showing GFAP in red and DAPI in blue. b) Cross‐section fluorescence imaging showing β‐tubulin III in green, GFAP in red and DAPI in blue for glial and neuronal spheroids at intermixing ratios of G80%/N20% and G20%/N80%. Scale bars = 100 µm. c) Radial localization frequency distribution of green‐β‐tubulin III, red‐GFAP and blue‐DAPI fluorescent signals according to their position relative to the center of the spheroid. The β‐tubulin III signal appears more frequently along the periphery of the spheroid cross‐section (*R/R0*≈1), whereas the GFAP and DAPI signals are more evenly distributed. *n* = 5 for G80%/N20% and *n* = 3 for G50%/N50%, with each cross‐section providing 500–1000 analyzed reference fluorescent points.

In order to confirm the cell sorting arrangement inferred from the spheroid 3D reconstruction, we visualized the fluorescent distribution of β‐tubulin III, GFAP and cell nuclei (DAPI) in the core of the spheroids. For this purpose, thin (20 µm) cross‐sections were obtained from the inner central planes of each spheroid, and subsequently stained for these markers (**Figure** **3**a). In G100% spheroids, GFAP‐labeled glial cells can be seen evenly distributed throughout the cross‐section area (Figure 3a). However, when neurons were included in the intermixed cell population, the localization of the red‐stained glial cells was observed mostly in the center of each spheroid plane, whereas the green‐stained neuronal cells were observed for the most part at the periphery. This arrangement was remarkably similar for both of the intermixing ratios of G80%/N20% and G20%/N80% (Figure 3b). Additional fluorescence staining images of spheroid cross‐sections showing this cell arrangement are provided in Figure S8 (Supporting Information). Cross‐sections of later spheroid maturation days for the G80%/N20% condition show a significant increase in the presence of β‐tubulin III‐green signal at the spheroid periphery, with a much higher signal intensity confirming the previously observed highly dense neuronal network (Figure 2b). Direct radial localization quantification of the fluorescent intensities of both β‐tubulin III, GFAP and DAPI was further assessed to elaborate on this hypothesis. This was done by dividing the spheroid fluorescent planar image using a micrometer‐sized grid, and then quantifying the distance between each of the section points within the grid and the center of the spheroid in order to obtain the position of each cell population relative to the spheroid center, given by the ratio of *R/R0*. (see Experimental Section). Results of this analysis evidenced a marked presence of green‐β‐tubulin III signal along the spheroid periphery (*R/R0*≈1), with red‐GFAP and blue‐DAPI signals having a more widely distributed position relative to the spheroid center (Figure 3c).

**Figure 4:**
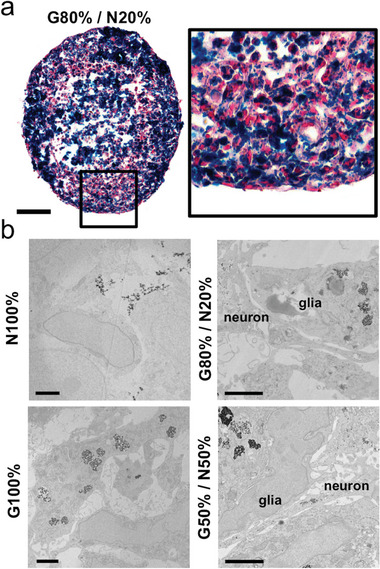
Spheroid Prussian blue staining and transmission electron microscope imaging analysis of magnetic nanoparticle internalization. a) Prussian blue staining showing the presence of nanoparticles inside the cells, in the form of blue spots suggesting a localization within endosomal compartments. Scale bar = 100 µm. b) Electron microscopy images showing nanoparticles localized near the cell membrane for the N100% condition, or in endosomal compartments in the G100% one. The glia and neuron labels in the G80%/20% and G50%/N50% conditions were inferred from these previous observations of nanoparticle localization. Scale bars = 2 µm.

We then proceeded to image the iron oxide nanoparticles internalization by the cells after magnetic labeling. Prussian blue staining of spheroid cross‐sections revealed the presence of the blue signal characteristic of iron throughout the whole spheroid, confirming the presence of nanoparticles inside individual cells (**Figure** **4**a). In addition, punctual blue iron signal shows the presence of nanoparticles inside endosomal compartments within the cells. This observation was further confirmed by transmission electron microscopy (TEM) imaging, showing the presence of groups of nanoparticles within cell endosomal compartments (Figure 4b). Overall, the glial cells appeared to internalize the nanoparticles more readily and efficiently, with many of them found densely confined within endosomes. In contrast, for neurons (N100%), the nanoparticles mostly appeared near the cell membrane. Additional images that confirm the observed nanoparticle localization in glial and neuronal spheroids are provided in Figure S9 and Figure S10 (Supporting Information). In the case of neuronal cells, rare endocytosis events were observed at later spheroid maturation time points, yet the distribution of nanoparticles remained mostly near the cell membrane (Figure S11, Supporting Information). Control TEM imaging of unlabeled cells are additionally provided for reference (Figure S12 and S13, Supporting Information). Thus, after intermixing, glial cells can be systematically identified by the presence of magnetic endosomes (Figure S14 and S15, Supporting Information). Neuronal axonal structures can be observed in the vicinity of glial cells for both of the two conditions of intermixing ratios imaged, with possible synapses being present (Figure S16, Supporting Information). Furthermore, qualitative TEM image analysis also led to the observation that the neuronal cells were more likely to be found at the periphery of the spheroid (Figure S17, Supporting Information).

**Figure 5:**
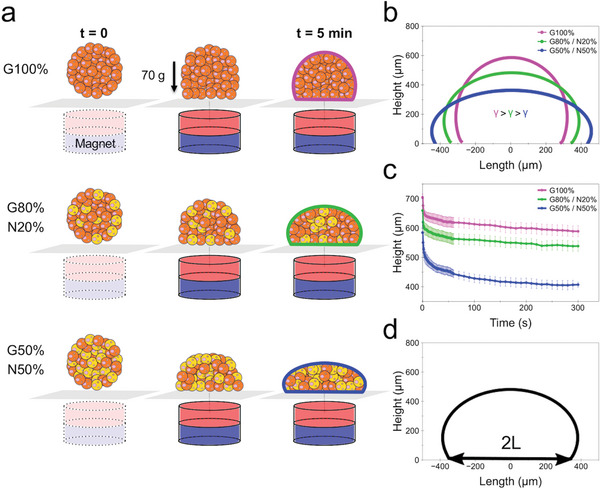
Measurement of the surface tension and elasticity in magnetic spheroids. a) Diagram showing the followed methodology. A single spheroid is placed on top of a glass coverslip. A magnet is then approached from below in a finely controlled manner and made to be in contact with the glass coverslip, directly below the spheroid position. Upon the applied magnetic field, the spheroid experiences a compression force in the range of 70 g, reaching an equilibrium shape in ≈5 min. b) Typical spheroid compression profiles for intermixed glial and neuronal cell populations, from which the surface tension γ is estimated by solving for Laplace capillary equations. c) Spheroid height change quantification upon application of the magnetic field over the course of the 5 min of compression. Data represent mean ± SEM; *n* = 8 for G100% and G80%/N20%; *n* = 6 for G50%/N50%. d) Elasticity *E* calculated from the lateral length contact area (2*L*) of the spheroid compression profile at equilibrium, as per Hertz's contact theory for an elastic sphere.

The next step was to exploit the magnetic fingerprint of the spheroids to determine how the different neuronal and glial intermixing ratios would affect the spheroid mechanical properties of surface tension and elasticity. The methodology is shown in **Figure** **5**a. It is based on the quantification of the progressive morphological change of the spheroid (i.e., its progressive flattening) when submitted to a magnetic force, until it reaches an equilibrium shape. Briefly, each spheroid is placed on top of a glass coverslip mounted inside an experimentation chamber that permits lateral optical imaging. Then, a magnet with a known field gradient is mechanically approached from below the chamber to direct external contact with the glass coverslip (Figure S18, Supporting Information; see Experimental Section). The force experienced by the spheroid as the magnet approaches thus induces a non‐destructive compression deformation along the vertical axis of the spheroid. With the spheroid magnetic moment in the range of 100 µemu (10^−7^ Am^2^) and the gradient generated by the magnet of 170 T m^−1^, the magnetic force (*M_v_gradB*) acting at the spheroid level is in the range of 15–20 µN. Importantly, it is a volume force of ≈5 × 10^4^ N m^−3^, similar to gravity and equivalent to 70 g. Such magnetic‐induced gravity is sufficient to flatten the spheroids without damaging the cells, being in the range of force experienced by cells when under a routine centrifugation step. Figure 5b shows the typical spheroid compression profiles under the applied uniform magnetic field for the glial and neuronal cell intermixing ratios of G100%, G80%/N20%, and G50%/N50%. After 5 min of magnetic field application the spheroid compression reaches an equilibrium profile, from which the surface tension γ can be calculated by solving for Laplace capillary equations. Naturally, a higher spheroid compression, i.e., a decrease in overall spheroid height, translates to a lower tissue surface tension. The quantification of the change in spheroid height during the 5 min of applied magnetic field is shown in Figure 5c. The G100% spheroids show an overall height loss of 115 µm on average, whereas the G80%/N20% and G50%/N50% ones decrease by 120 and 180 µm on average, respectively. We additionally estimated the spheroid elasticity *E* from the spheroid compression profile at equilibrium following Hertz's contact theory for an elastic sphere, calculated from the lateral length contact area (2*L*) of the spheroid (Figure 5d), given the known variables of spheroid radius, magnetization, volume and magnetic field gradient (see Experimental Section).

**Figure 6:**
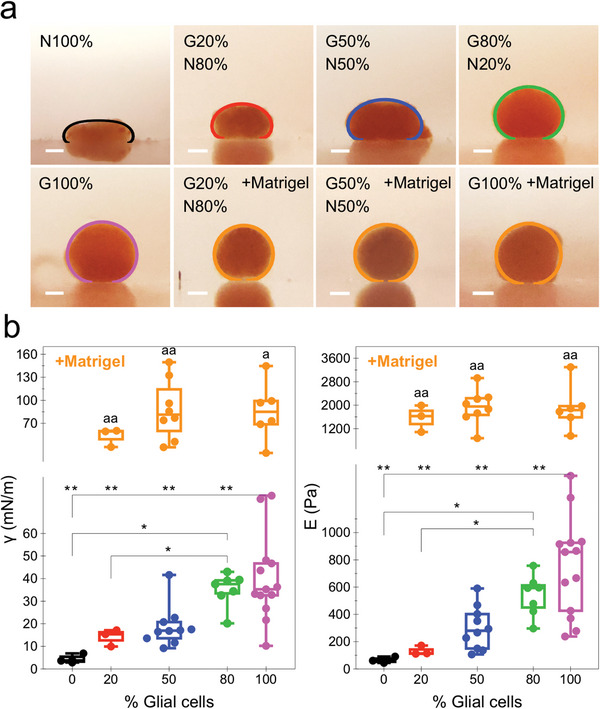
Spheroid surface tension and elasticity dependence on the glia/neuron cell population ratio. a) Lateral optical imaging of spheroids for all measured cell intermixing ratio conditions with the corresponding equilibrium shape fit obtained after 5 min of compression under the applied magnetic field. b) Box plots showing the spheroid surface tension and elasticity quantification for spheroids at day 1 of maturation and at intermixing ratios of N100%, G20%/N80%, G50%/N50%, G80%/N20%, and G100%, as well as the G50%/N50% and G100% ratio spheroids pre‐mixed with Matrigel matrix. *n* = 3, except for groups where glial cells ≤ G20%, where *n* = 2; *p < 0.05, **p < 0.01; ^a^ *p* < 0.05, ^aa^ *p* < 0.01 versus same cell ratio condition without Matrigel. Scale bars = 200 µm.

**Figure** **6** demonstrates the dependence of the surface tension and elasticity on the ratio of glial and neuronal cell populations for spheroids at day 1 of maturation. Figure 6a shows the typical optical lateral imaging of the spheroids under magnetic compression for all the conditions tested, along with the spheroids’ final equilibrium profile after 5 min of compression. Images of spheroids before application of the magnetic field gradient, as well as additional images of compression profiles are provided in Figure S19 and S20 (Supporting Information), respectively. It can be visually observed that as the number of glial cells in the spheroid composition increases, the lower the compression is, or conversely, the higher the surface tension of the spheroid. The compression effect is mitigated by the addition of Matrigel into the mixture, more remarkably so for the spheroids with fewer neuronal cells. The quantification of the surface tension γ and the elasticity *E* thus revealed that both of these biomechanical parameters are proportional to the percentage of glial cells in the spheroid composition (Figure 6b), with the median surface tension γ for the N100%, G20%/N80%, G50%/N50%, G80%/N20%, and G100% equal to 4, 15, 17, 37, and 35 mN m^−1^, respectively. The median elasticity *E* was found to be at 60, 114, 280, 593, and 857 Pa for the respective spheroid composition conditions. The addition of Matrigel into the cell mixture significantly increased the surface tension and elasticity for all conditions.

**Figure 7:**
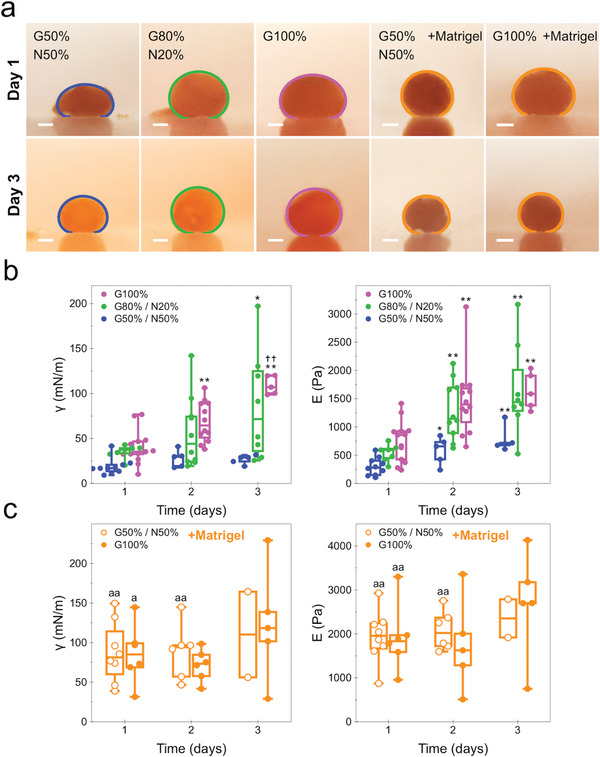
Spheroid surface tension and elasticity dependence on the glia/neuron cell population ratio over 3 days of maturation. a) Lateral optical imaging of spheroids for all measured cell intermixing ratio conditions with the corresponding equilibrium shape fit obtained after 5 min of compression. Images show spheroids at day 1 and day 3 of maturation. b) Box plots showing spheroid surface tension and elasticity quantification for spheroids at these maturation time points and for intermixing ratios of G50%/N50%, G80%/N20%, and G100%. c) Box plots showing the surface tension and elasticity of G50%/N50% and G100% ratio spheroids pre‐mixed with Matrigel matrix. *n* = 3; *n* = 2 for later maturation time point spheroids with Matrigel; *p < 0.05, **p < 0.01 versus day 1; ^††^ *p* < 0.01 versus day 2; ^a^ *p* < 0.05, ^aa^ *p* < 0.01 versus same cell ratio condition without Matrigel, at same day of maturation. Scale bars = 200 µm.

Next, we extended this analysis to further spheroid maturation stages, with similar results. For the conditions analyzed, the spheroids’ final equilibrium profile after 3 days showed an overall lower compression, an indication of an increase in tissue cohesivity (**Figure** **7**a). Naturally, such a change in the equilibrium profile translates to an increase in surface tension, and a decrease in the contact area 2*L*, hence a higher elasticity. Indeed, the surface tension showed a median increase for all the ratio conditions (Figure 7b). For the G50%/N50% spheroids, the surface tension increased from 17 to 29 mN m^−1^ after 2 days of maturation, albeit it being found to be not significant. On the other hand, spheroids at G80%/N20% showed a significant increase after day 3, from 37 up to 71 mN m^−1^. The neuron‐less condition G100% increased from 35 up to 64 and 107 mN m^−1^ for day 2 and 3 of maturation, respectively. The elasticity parameter saw similar results, with overall significant increases compared with the initial day 1 of maturation, with G50%/N50% spheroids reaching a maximum elasticity of 702 Pa, whereas the G80%/N20% and G100% reached values of 1438 and 1585 Pa at day 3, respectively. The addition of Matrigel further increased both biomechanical parameters, although the changes were only significant at day 1, and at day 2 only for the G50%/N50% spheroid condition (Figure 7c).

## 3 Discussion

We validated an all‐in‐one magnetic based strategy for brain‐like tissue formation, maturation, and mechanical stimulation. We used magnetically‐labeled primary glial and neuronal cells intermixed at different ratios. Both cell types show the capacity of nanoparticle uptake, yet glial cells appear to more readily internalize and endocytose them compared to neuronal cells, probably due to the fast, 5 min labeling procedure followed. The nanoparticle labeling process thus confers each cell with a magnetic moment. Current research advances have taken advantage of such cell magnetization to control neuronal outgrowth under magnetic fields.^[^ ^26^, ^27^ ^]^ Here, it is thanks to this cell magnetization process that they can be aggregated under an external magnetic field within minutes, rapidly adopting a spherical shape in the process. Mature spheroids are obtained in less than 1 day, permitting the direct observation of important early stage tissue morphogenesis processes that were otherwise unattainable with non‐magnetic spheroids produced with the typical centrifugation into agarose molds method, probably due to a less overall cohesivity conferred from the initial aggregation force translating into longer maturation requirements. In the produced magnetic spheroids, the arrangement of the cells on the outer layers of the structure changes drastically between the first and eighth days of maturation, as neuronal cells extend their dense axonal network and spread over the glial cell population, which remain mostly confined in the center of the spheroid. Neurite length may reach ≈100 µm after day 1, which exceeds the length observed in 2D cultures in astrocyte conditioned medium on Matrigel.^[^ ^28^ ^]^ This highlights the fact that our culture conditions promote healthy, fast growing neurons.

The idea of the envelopment of one cell population over another as they become intermixed was initially described by the differential adhesion hypothesis, which states that the surface tension, itself a result of the cohesive and adhesive forces between the component cells, bestows the tissue its liquid‐like behavior.^[^ ^29^ ^]^ It is therefore the surface tension, or the intensity of cohesion among the cells, that drives the envelopment of one cell population over another. Thus, the cell population with the lower surface tension tends to spread over or envelop the one with the higher surface tension.^[^ ^30^ ^]^ The mechanics of such tissue behavior and organization are typically coordinated by cadherins, which regulate cell‐cell adhesion through linkage with the actin cytoskeleton, as well as cell‐extracellular matrix signaling cues regulated by integrins.^[^ ^31^ ^]^ As such, tissue sorting in embryogenesis has been linked to differing cadherin concentrations,^[^ ^32^ ^]^ coupled with contractile forces and more complex signaling pathways, such as Ephrin signaling.^[^ ^33^ ^]^ Similarly, it has been evidenced in vitro that cell populations with higher concentrations of cadherins possess a higher surface tension and are thus enveloped by cell populations with lower overall cadherin expression.^[^ ^34^ ^]^ Here, we show a similar behavior with pure glial and pure neuronal cell spheroids, with the former expressing significantly higher levels of E‐cadherin, suggesting a higher surface tension for this cell population.

Building on this previous knowledge, we then show that primary neuronal cells sort themselves around glial cells after cell intermixing and magnetic aggregation into a spheroid, with this specific architecture being maintained over a period of 8 days of maturation as the neuronal axonal network spreads and extends along the spheroid periphery. In order to further prove this concept, we sought out to elucidate the specific contribution of the glial and neuronal cells to the overall spheroid surface tension. This biomechanical property is obtained by subjecting the spheroid tissue to a controlled force and allowing it to reach an equilibrium shape,^[^ ^35^ ^]^ which is reached as the applied force is balanced out by the tissue cohesive forces driving its spherical shape conformation. In this work, the surface tension was measured using a novel technique that exploits the magnetic fingerprint of the spheroid due to the labeling of cells with magnetic nanoparticles: by subjecting the spheroid to an external field gradient, it experiences a deformation along its vertical axis and reaches the equilibrium shape within minutes. The surface tension measurements of the glial and neuronal spheroids at different intermixing ratios correlates with the observed cell sorting behavior, with results indicating that the increase in spheroid surface tension is proportional to the number of glial cells in the mixture. On the other hand, the presence of neuronal cells tends to slow down the time‐dependent increase of γ. Thus, in a similar fashion described by the differential adhesion hypothesis, the lower surface tension of the neuronal cells could be acting as a driver of their spreading and arrangement over the glial cells in the early stages of the spheroid formation process (< day 1). Although there exist scarce or no reports in the literature of the surface tension of primary brain cells, this parameter has been studied in glioblastoma aggregate models, with the surface tension found in the range of 7–46 mN m^−1^ depending on the tumoral cell line,^[^ ^36^, ^37^ ^]^ and similar to our reported value of 35 mN m^−1^ for the G100% spheroids. The addition of Matrigel significantly increased the surface tension for the G20/N80% and G50%/N50% ratio conditions compared to the spheroids without matrix, at 3.7‐ and 4.6‐fold at day 1 of maturation. This effect is explained by an increase in spheroid cohesivity by the addition of a matrix. The surface tension increase due to the addition of Matrigel in the G100% spheroids was overall lower, at 2.4‐fold at day 1. This suggests that the surface tension in glial tissue alone, being the highest between all the measured conditions, possesses a threshold cohesivity that cannot be further increased by external inputs.

We further took advantage of the equilibrium shape reached after magnetic compression of the spheroids to infer their elastic properties. For the G50%/N50% ratio condition, which more closely resembles the brain composition, the elasticity was found to be ≈280–700 Pa depending on the spheroid maturation time. These values are in agreement with previously reported elastic moduli in spheroids with a similar composition, at 100–200 Pa,^[^ ^38^ ^]^ and that of bulk murine hippocampal tissue, found to be in the 300–660 Pa range by scanning^[^ ^39^ ^]^ and atomic force microscopy.^[^ ^40^ ^]^ Interestingly, it is at this specific substrate elastic modulus range where neuronal differentiation and β‐tubulin III expression both peak.^[^ ^11^ ^]^ The addition of Matrigel into the cell mixture increased spheroid elasticity by 11.8‐fold in G20%/N80%, 6.5‐fold in G50%/N50% spheroids and 2.5‐fold in G100% spheroids at day 1. This is expected, given that Matrigel alone possesses an elastic modulus of ≈450 Pa.^[^ ^41^ ^]^ Our elasticity measurements confirm that, at the fundamental level of neuronal and glial cells intermixing, the brain‐like tissue spheroids remain relatively soft in comparison with other types of tissues. Indeed, both these types of cells have been evidenced to be relatively soft when measured individually, with glial cells found to be softer than neurons.^[^ ^39^ ^]^ In contrast to this, we measured a higher elasticity for pure glial spheroids. This suggests that the mechanical properties at the spheroid tissue level do not reflect the properties of individual cells. For instance, astrocytes are capable of expressing fibronectin^[^ ^42^ ^]^ and laminin,^[^ ^43^ ^]^ which may induce a reinforcement of adhesive contacts with neurons and lead to a higher elastic modulus.

## 4 Conclusion

To the best of our knowledge, this is the first report of a viable reconstitution of spheroids from intermixed primary glial and neuronal cells after a rapid magnetic aggregation step. The resulting magnetically active brain‐like tissue spheroids can thus be controlled by remote magnets. We used this magnetic fingerprint to retrieve the biomechanical properties of surface tension and elasticity from a magnetic‐driven deformation. Remarkably, we evidenced a transition from very low surface tension to much higher ones by the increase of the proportion of glial cells in the neuron‐glia mix. Not only this is the first determination of the important difference in the individual contribution of glial and neuronal cells to the overall tissue surface tension, but it additionally evidences the impressive local organization observed at the spheroid level, with neurons sorting out to the spheroid periphery. The 3D brain tissue model recapitulates some of the physiology of the cortex, and provides insight into the predetermination of cell population sorting and arrangement with the surface tension as a likely contributor, recalling self‐organization phenomena at work more generally in tissue embryogenesis.

## 5 Experimental Section

### In Vivo Extraction of Neuronal and Glial Cells

Cortical neurons and glial cells were harvested from the cortical hemispheres dissected from C57BL/6 mouse embryo brains at embryonic day 15‐16 (Charles River Laboratories). Pregnant mice were euthanized by cervical dislocation, and the embryos were surgically removed and euthanized by a spinal cord incision at the neck level before brain extraction. The procedure was carried out three independent times, each one approximately yielding 5–6 embryo brains. After extraction of both cortical hemispheres, they were rinsed thoroughly using Gey's Balanced Salt Solution (GBSS, Thermofisher), and then dissociated in Dulbecco's Modified Eagle's medium (DMEM, Thermofisher) containing 25 mg mL^−1^ of papain (Sigma‐Aldrich) during 10 min at 37 °C. The enzymatic dissociation was halted by the addition of 10% fetal bovine serum (FBS, Thermofisher). Samples were then dissociated in DMEM medium supplemented with DNase I (Sigma‐Aldrich) with the aid of a pipette. The harvested cells were then centrifuged at 80 g for 6 min and resuspended in DMEM medium supplemented with 5% FBS, 1% N‐2 (Thermofisher), 2% B‐27 (Thermofisher) 2 mM GlutaMAX (Thermofisher), 1 mM sodium pyruvate (Thermofisher) and 20 µg mL^−1^ gentamicin (Thermofisher), henceforth denoted as DMEMc. All experimental handling of neuronal cells was performed in suspension, right after collection, and processing.

For glial cell extraction and culture, the cortical hemispheres were rinsed in GBSS and then transferred to a petri dish previously coated with 15 µg L^−1^ of Poly‐L‐ornithine (Sigma‐Aldrich). Each cortex was then cut into small pieces in Modified Eagle's Medium (Thermofisher) supplemented with 10% FBS, 2 mM GlutaMAX, 1 mm sodium pyruvate and 20 µg mL^−1^ gentamicin. Tissue pieces were mechanically dissociated by vigorous pipetting in the culture dish, and the obtained cell suspension was incubated for one week at 37 °C in a humidified incubator at 5% CO_2_, changing the culture medium every 3 days. Before magnetic labeling, the glial cells were detached with 1X TrypLE™ Express Enzyme (Thermofisher) after a 10 min incubation at 37 °C.

### Magnetic Labeling of Cells

The magnetic nanoparticles used for cell labeling were produced using the standard procedure by co‐precipitation of iron salts. Briefly, FeCl_3_ (3 g), and FeCl_2_ (8.1 g) were diluted in 5 mL deionized (DI) water with 25% ammonium hydroxide used as a precipitating agent (pH 10). After 20 min of heating at 90 °C, the solution was decanted on magnet, washed with acetone and H_2_O, centrifuged at 8000 rpm for 10 min and re‐dispersed in 10 mL of DI water. Citrate (2 g in 20 mL DI water) was added, and the resulting solution was heated at 90 °C for 1 h under vigorous agitation, resulting in both nanoparticle oxidation and citrate chelation to ensure colloidal stability. The nanoparticle diameter was 8 ± 1.7 nm, as estimated by TEM.

For magnetic labeling, the neuronal and glial cell populations were both counted and intermixed in DMEM medium (without serum) at the corresponding ratios. Mixed populations were then centrifuged at 100 g for 5 min and resuspended in serum‐free RPMI medium (Gibco) with magnetic nanoparticles at [Fe] = 50 mM and supplemented with citrate at 5 mM. The cells were magnetically labeled in this solution during 5 min at 37 °C in a humidified incubator at 5% CO_2_ and while under shaking motion.

### Magnetic Molding of Spheroids

The spheroids were formed using the magnetic molding method.^[^ ^44^ ^]^ Briefly, 2% agarose (A0576, Sigma‐Aldrich) in phosphate buffered saline (PBS) was used to create molds in a 22.1 cm^2^ petri dish (TPP). The molds’ spherical shape was defined by partially immersing 1 mm steel beads (CIMAP) in the liquid agarose and holding them in place using an array of 6 × 2 mm cylindrical neodymium magnets (Supermagnete). The steel beads were removed with the aid of a magnet after agarose gelation, and the dishes with the molds were then sterilized by UV exposition for 30 min and filled with PBS before use. Immediately after magnetic labeling, the cells were centrifuged at 100 g for five minutes and each condition of mixed neuronal and glial cells was then resuspended in 50 µL of DMEMc, mixing thoroughly with the aid of a pipette to break out aggregates of cells. Using the same array of neodymium magnets placed below the dish and aligned with the agarose molds, 7 µL of the magnetic cell suspension (≈100000 cells) were pipetted at the liquid interface on top of the spherical molds, resulting in the cells being magnetically attracted toward the direction of the gradient, hence filling up the spherical agarose mold in the process. Cell aggregation after seeding was rather fast, forming a visible spheroid in under 3 min. The array of magnets was then removed and the PBS was carefully aspirated from the dishes and replaced with DMEMc, and the formed spheroids embedded in the molds were left to mature overnight at 37 °C in a humidified incubator at 5% CO_2_. The next day, the spheroids were collected with the aid of a cut 1 mL pipette tip and subsequently incubated in Neurobasal Medium (Thermofisher) supplemented with 1% FBS, 2% B‐27, 1% L‐glutamine (Thermofisher) and 1% sodium pyruvate.

For the formation of spheroids containing Matrigel (Corning) matrix, the initial cell population was mixed with ≈40% of Matrigel solution in volume before pipetting into the molds. All Matrigel and cell handling procedures were performed at 4 °C to avoid gelation of the matrix. ≈14 µL of the cell/matrix suspension were slowly pipetted into the molds.

### Spheroid Magnetization Quantification

The magnetization of individual spheroids was quantified using a vibrating sample magnetometer (Quantum Design, Versalab), with each measurement performed at 300 K over a range of 0–3 T. Data shown refers to bulk magnetization expressed in µemu.

### Spheroid Cryosectioning and Prussian Blue Staining

After fixation in paraformaldehyde, cryosectioning of the spheroids and Prussian blue staining were carried out by the Plateforme Histologie, Immunomarquage, Microdissection laser (HistIM, Institut Cochin, Paris, France). The obtained cross‐sections possessed a thickness of 20 µm and were mounted on a glass microscope slide.

### Immunofluorescence Staining

At the desired maturation time point, spheroids were fixed in 4% paraformaldehyde for 1 hour at room temperature. They were then washed in PBS and permeabilized in 0.1% Triton X‐100 in PBS for 15 min at room temperature, and then subsequently blocked in a solution consisting of 2% bovine serum albumin solution in PBS with 0.1% Triton X‐100 for 3 h at room temperature. Spheroids were then incubated in anti‐GFAP primary antibody (G4546, Sigma‐Aldrich) at a 1:200 dilution and anti‐β‐tubulin isotype III primary antibody (T5076, Sigma‐Aldrich) at a 1:500 dilution in blocking solution at room temperature overnight. The spheroids were then washed thoroughly in PBS and incubated in Alexa Fluor 594‐conjugated goat anti‐rabbit IgG secondary antibody (A11037, Thermofisher) at a 1:500 dilution in blocking solution and Alexa Fluor 488‐conjugated goat anti‐mouse IgG secondary antibody (A11029, Thermofisher) at a 1:400 dilution at room temperature overnight. Lastly, after washing with PBS, spheroids were incubated in a 300 nM DAPI solution (D3571, Invitrogen) at a 1:500 dilution in PBS at room temperature overnight. A spheroid z‐stack section of ≈200 µm was imaged using a Leica DMi8 inverted confocal microscope (Leica Microsystems) using a 25x water immersion objective.

For cross‐section immunofluorescence staining, the obtained cryosections were stained as described above, with primary and secondary antibody incubations both reduced to 2 h at room temperature. Cross‐sections were then mounted using Fluoromount Aqueous Mounting Medium (F4680, Sigma‐Aldrich). Imaging was performed after a 2 h gelation of the mounting medium.

### Image Analysis and Fluorescence Quantification

Image analysis and 3D spheroid reconstruction were done using the ImageJ open software. For the analysis of the localized radial fluorescent intensity of the neuronal and glial cell populations, each image cross‐section was divided in 40 × 40 µm sections. Each of the section points was then assigned an x,y coordinate. Using a custom Python code, each set of coordinates was then individually compared to the x,y coordinates of the spheroid center (manually set) in order to obtain the *R/R0* ratio, where *R* denotes the distance from each section point to the x,y coordinates of the spheroid center, and with *R0* denoting the radius of the spheroid from its center to the closest point of the periphery along the analyzed x,y coordinates of the section point. Thus, the *R/R0* ratio directly measures how far each data point was from the spheroid center. This analysis was performed for each section of the grid comprising the complete spheroid in the image, and for all three fluorescence channels imaged (green for β‐tubulin III, red for GFAP and blue for DAPI).

### Transmission Electron Microscope Imaging

Spheroids were fixed in 2% glutaraldehyde in 0.1 M cacodylate buffer (Sigma‐Aldrich) for 1 h at room temperature. Individual spheroids were then contrasted with 0.5% oolong tea extract and post‐fixed in 1% osmium tetroxide (Sigma‐Aldrich) and 1.5% potassium cyanoferrate (Sigma‐Aldrich). Samples were then gradually dehydrated in graded ethanol, at steps from 30% to 100% ethanol, and subsequently embedded in Epon resin. Thin slices of ≈70 nm were imaged using a Hitachi HT 7700 transmission electron microscope operated at 80 kV.

### Surface Tension and Elasticity Quantification

In order to obtain the surface tension measurement at the spheroid level, spheroids were placed inside a chamber filled with phenol‐free cell medium and possessing two microscope glass slides on its sides to permit imaging, as well as an additional glass coverslip at the bottom of the chamber. A cylindrical 6 × 6 mm neodymium magnet (Supermagnete), generating a uniform magnetic field gradient of *gradB* = 170 T m^−1^ within a cylindric volume of 2 mm in height and 2 mm in diameter, was then approached with fine precision using a vertical lift stage (X‐VSR20A, Zaber Technologies Inc.) and made to come in contact with the glass coverslip at the bottom of the chamber. The full system was placed inside a custom‐built thermoregulated chamber supplemented with CO_2_ (Microscope Heaters–Digital Pixel Limited, Brighton, United Kingdom). As each spheroid possesses a magnetic fingerprint conferred by the labeling with magnetic nanoparticles, they experience a compression deformation in the direction of the magnetic field gradient as the magnet approaches and touches the glass coverslip beneath. The spheroids’ lateral compression profiles were monitored live with a Canon EOS R6 digital camera coupled with a Canon MP‐E 65 mm f/2.8 1–5x Macro lens until equilibrium compression shape was reached (≈5 min). The surface tension parameter can then be calculated from the spheroid shape in equilibrium when compressed by integrating the Laplace law of capillarity^[^ ^45^ ^]^ to extract the capillary constant *c*:

(1) $$c\;=\;M_{v}\;gradB\;\gamma^{-1}$$

where *M*_v_ is the volume saturation magnetization of the spheroid, previously calculated through vibrating sample magnetometry, *gradB* the magnetic field gradient applied and γ the macroscopic surface tension of the spheroid.

The spheroid elasticity quantification was done following Hertz's contact theory for an elastic sphere, estimated by equation (2):

(2) $$L_{elastic}={\left[\left(1-\sigma^{2}\right)\pi /E\right]}^{1/3}f_{v}{\;}^{1/3}R^{1/3}$$

where 2*L* is the lateral length contact area of the spheroid when compressed, *σ* is the Poisson ratio, here set to ½, *f_v_* the volumetric force exerted on the spheroid by the magnetic field gradient, equal to *M_v_ gradB, R* the initial radius of the spheroid and *E* its elastic modulus.

### Statistical Analysis

Statistical analysis of multiple group comparisons was done using the one‐way analysis of variance, followed by a post‐hoc Tukey's test. Statistical analysis of between two groups was done using the Student's two‐Sample t‐test. All analyses were performed using the Matlab software (MathWorks Inc.). Data representation details and *n* number values were provided in each corresponding figure legend. Statistical significance were considered for values of *p* < 0.01 or *p* < 0.05.

### Ethics Approval Statement

The experimental use of laboratory mice was performed in accordance with the European Community guidelines of animal care, under the Institut Curie license #C75‐05‐18, 24/04/2012, reporting to “Comité d’Éthique en matière d'expérimentation animale Paris Centre et Sud” (National registration #59).

## Conflict of Interest

The authors declare no conflict of interest.

## Supporting information

Supporting Information

## Data Availability

The data that support the findings of this study are available from the corresponding author upon reasonable request.
