# Supplementary material for: Surface Tension and Neuronal Sorting in Magnetically Engineered Brain‐Like Tissue
Source: Adv Sci (Weinh). 2023 Aug 6;10(27):2302411. doi: 10.1002/advs.202302411 (PMC10520685; doi:10.1002/advs.202302411)
Supplement: Supplementary file 1 — Supporting Information [file ADVS-10-2302411-s001.pdf]

ADVANCED SCIENCE
Open Access

Supporting Information

for *Adv. Sci.*, DOI 10.1002/advs.202302411

Surface Tension and Neuronal Sorting in Magnetically Engineered Brain-Like Tissue

*Jose E. Perez, Audric Jan, Catherine Villard and Claire Wilhelm**

Surface Tension and Neuronal Sorting in Magnetically Engineered Brain-Like Tissue

*Jose E. Perez, Jan Audric, Catherine Villard and Claire Wilhelm**

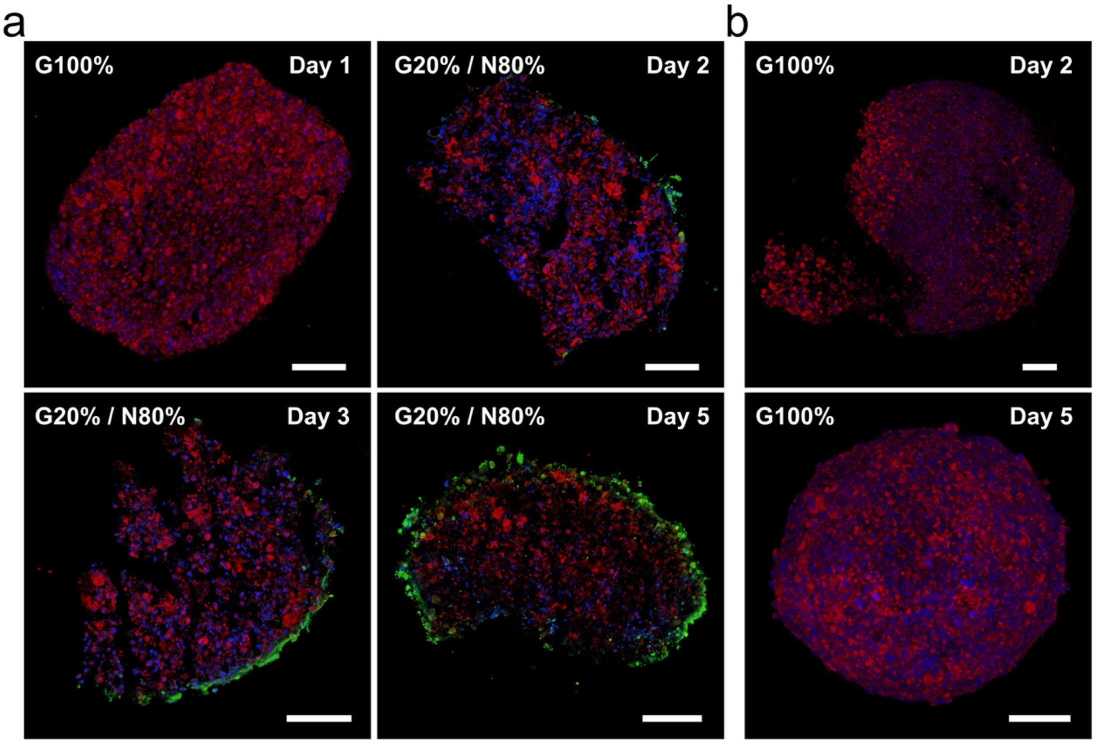

Figure S1. Fluorescence imaging of the neural stem cell marker SOX2. Images show SOX2 in green, GFAP in red and DAPI in blue, in both spheroid cross-sections (a) and G100% complete 3D-reconstructed spheroids (b). The neural stem cell marker SOX2 is absent from the isolated glial population, evidenced by the lack of green signal in both the G100% tissue cross-section and the complete G100% spheroids. On the other hand, the presence of this marker is observed in G20% / N80% spheroid cross-sections, with a higher signal observed after 5 days of spheroid maturation. These results evidence the survival of these progenitor cells for the isolated neuronal population. Cross-sections and complete spheroids were incubated with anti-SOX2 primary antibody (AF2018, R&D Systems), followed by an incubation with Alexa Fluor 488®-conjugated goat anti-mouse IgG secondary antibody (A11029, Thermofisher), following the same protocol described in the Methods section of the manuscript. Scale bars = 100 µm.

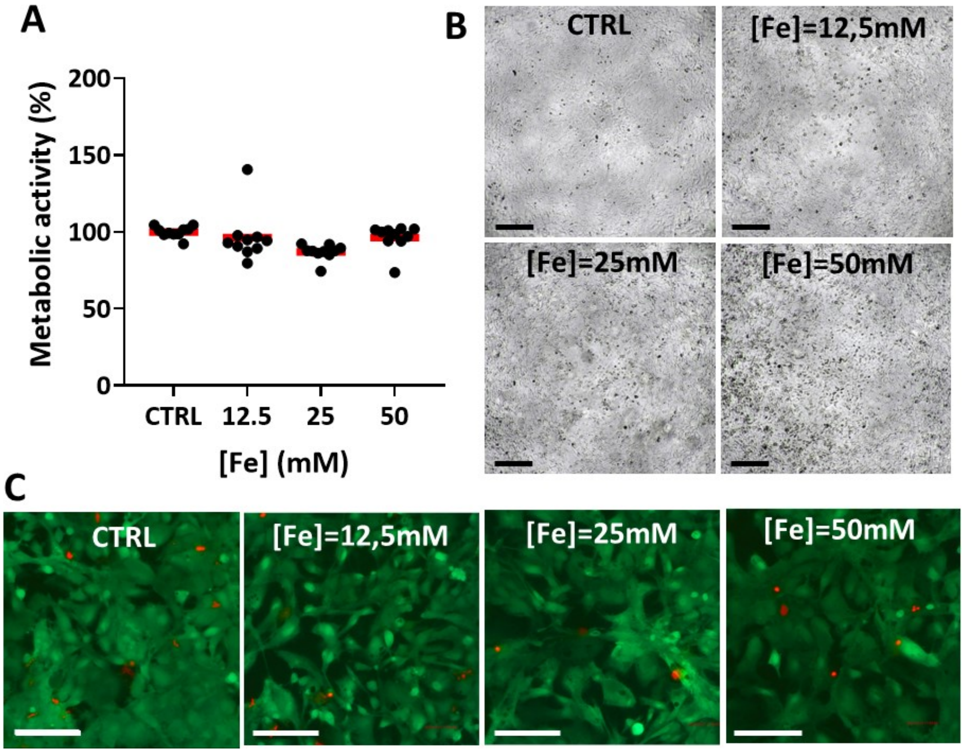

Figure S2. (A) Metabolic activity of primary glial cells measured 24 hours after magnetic labeling for 5 min at [Fe] = 12.5, 25, and 50 mM by Alamar Blue assay (Invitrogen) according to supplier's instructions. To do this, a 96-well plate was seeded with 50,000 glial cells per well after a 30 minutes coating with 15 µg/mL Poly-L-ornithine in phosphate buffered saline, one week after cell isolation. They were left to mature for 2 days before being labeled with the nanoparticles (12 wells per condition). 24 hours later, 100 µL of Alamar Blue reagent were added to each well and left to react for 2 hours at 37 °C. The fluorescence was then recorded at an excitation between 530–560 and an emission at 590 nm using an Ensight multi-mode plate reader (PerkinElmer). (B) Typical bright field images of the glial cells for each condition. Scale bars= 500 µm. (C) Live/Dead staining performed under the same conditions 24 hours after magnetic labeling and following supplier instructions (LIVE/DEAD™ Cell Imaging Kit R37601, Invitrogen). Typical fluorescent images (live cells in green, dead cells in red) were acquired by fluorescence microscopy (simultaneous blue and green excitation, color camera acquisition). Scale bars = 200 µm.

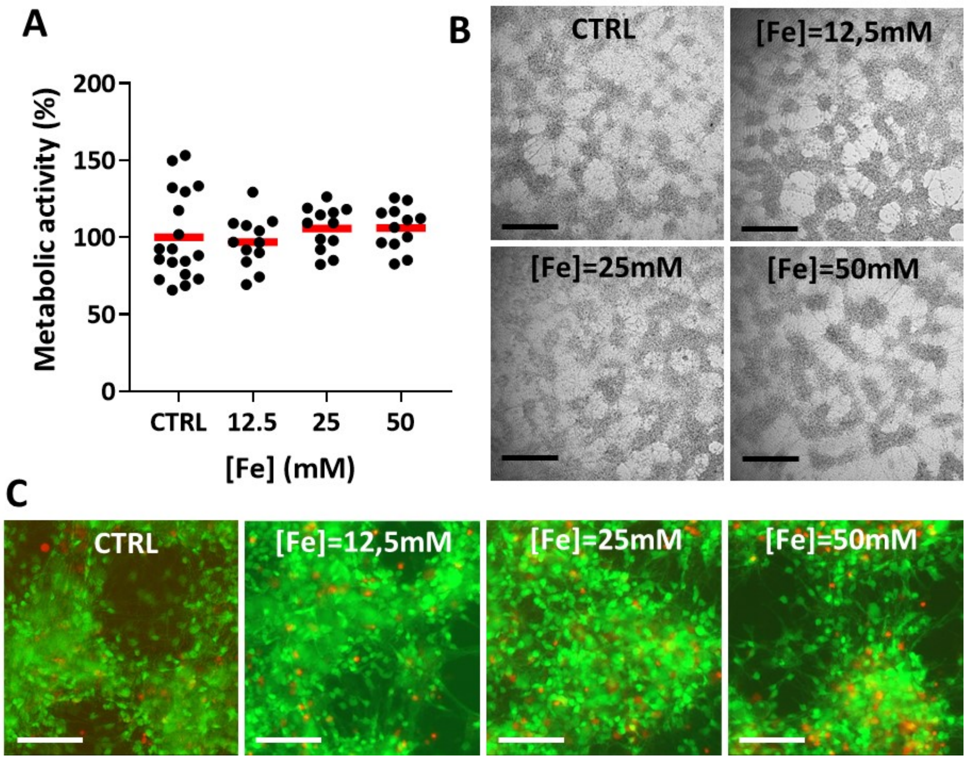

Figure S3. Cell viability analysis of primary neuronal cells under the same experimental conditions as shown in Figure S1. Magnetic labeling was performed after 3 days of maturation. (A) Metabolic activity. (B) Bright field images. Scale bars = 500 µm. (C) Live/Dead fluorescent staining. Scale bars = 200 µm.

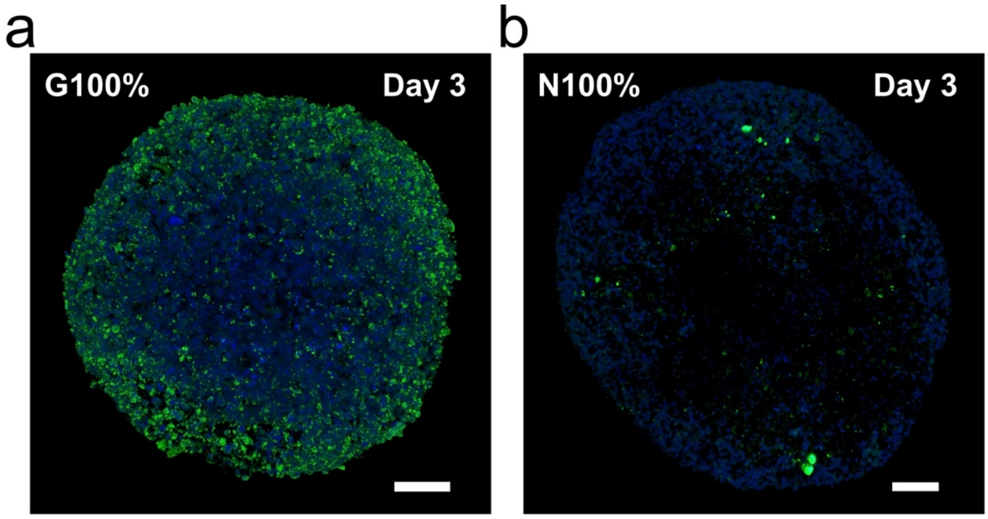

Figure S4. Fluorescence imaging 3D reconstruction showing the expression of E-cadherin in green and DAPI in blue. Spheroids were incubated with anti-E-cadherin primary antibody (SHE78-7, Invitrogen), followed by an incubation with Alexa Fluor 488®-conjugated goat anti-mouse IgG secondary antibody (A11029, Thermofisher), following the same protocol described in the Methods section of the manuscript. Scale bars = 100 μm.

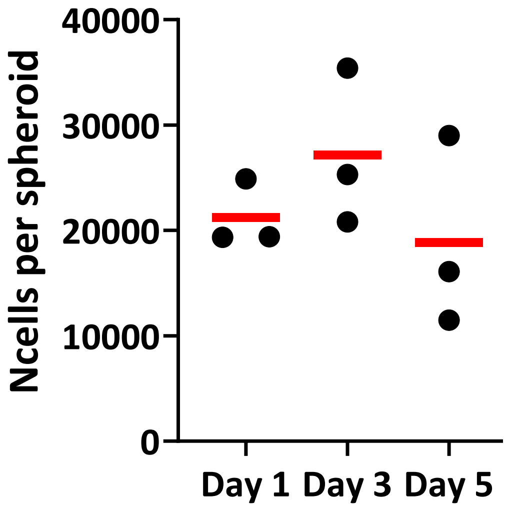

Figure S5. Cell quantification of G100% spheroids over a 5 days period, with each dot representing a single spheroid. Spheroids were placed in TrypLE™ Select Enzyme 10x (Thermofisher) for 30 minutes at room temperature to induce complete dissociation before cell counting.

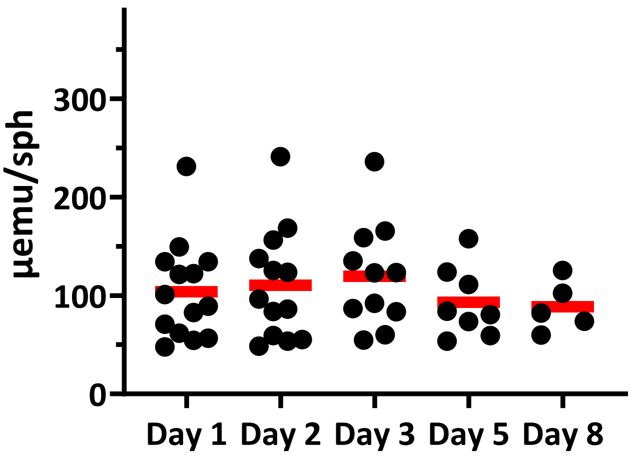

Figure S6. G100% spheroid magnetization quantification over 8 days of culture by vibrating sample magnetometry. Each dot represents a single spheroid measured. A slight decrease in magnetization in a non-significant way is observed after 5 days of culture.

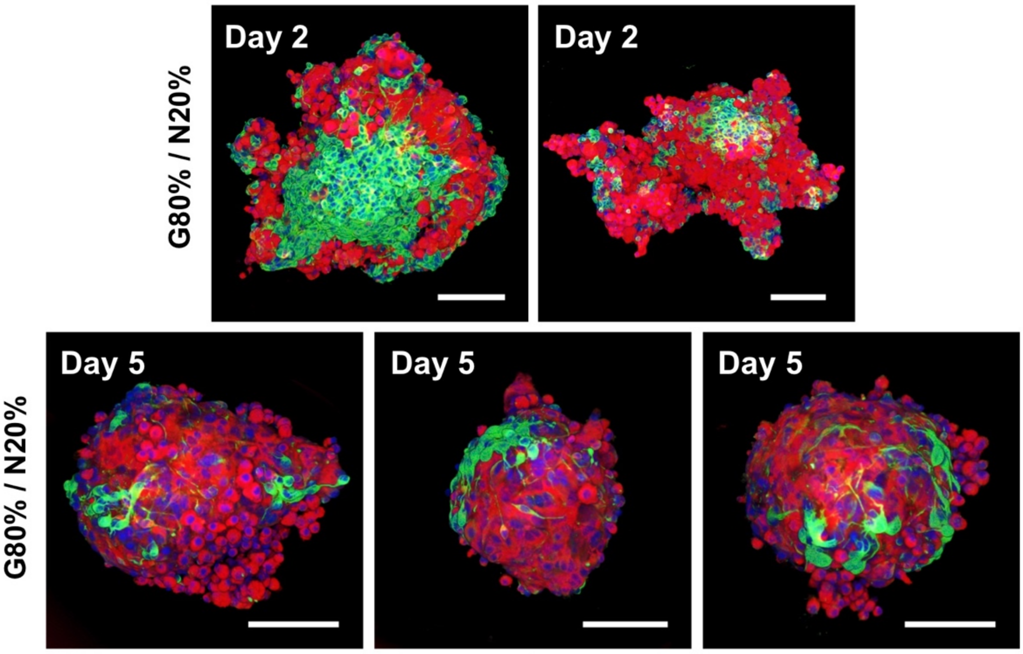

Figure S7. Primary glial and neuronal cell sorting in non-magnetic spheroids prepared by centrifugation in agarose molds. Briefly, 2% agarose in phosphate buffered saline was prepared in 96-well plates. Before agarose gelation, an in-house 3D printed device with spherical end-tips of approximately 500 µm was used to create spherical molds in the agarose. The device was then removed and the 96-well plate was sterilized in ultraviolet light for 30 minutes. Meanwhile, primary glial cells were pre-labeled with Dil tracer (D282, Invitrogen) at a concentration of 20 µg/mL for 1 hour at 37 °C before cell counting. Then, a mixture of 48,000 primary glial cells and 12,000 primary neurons (G80% / N20%) was placed in each well, followed by a single routine centrifugation step to ensure cell aggregation within the agarose mold. The aggregates were then left to mature for up to 5 days within the molds. Images show the fluorescence imaging 3D reconstruction at day 2 and day 5 of maturation, showing β-tubulin III in green, the Dil tracer signal of the glial cell population in red and DAPI in blue. Scale bars = 100 µm.

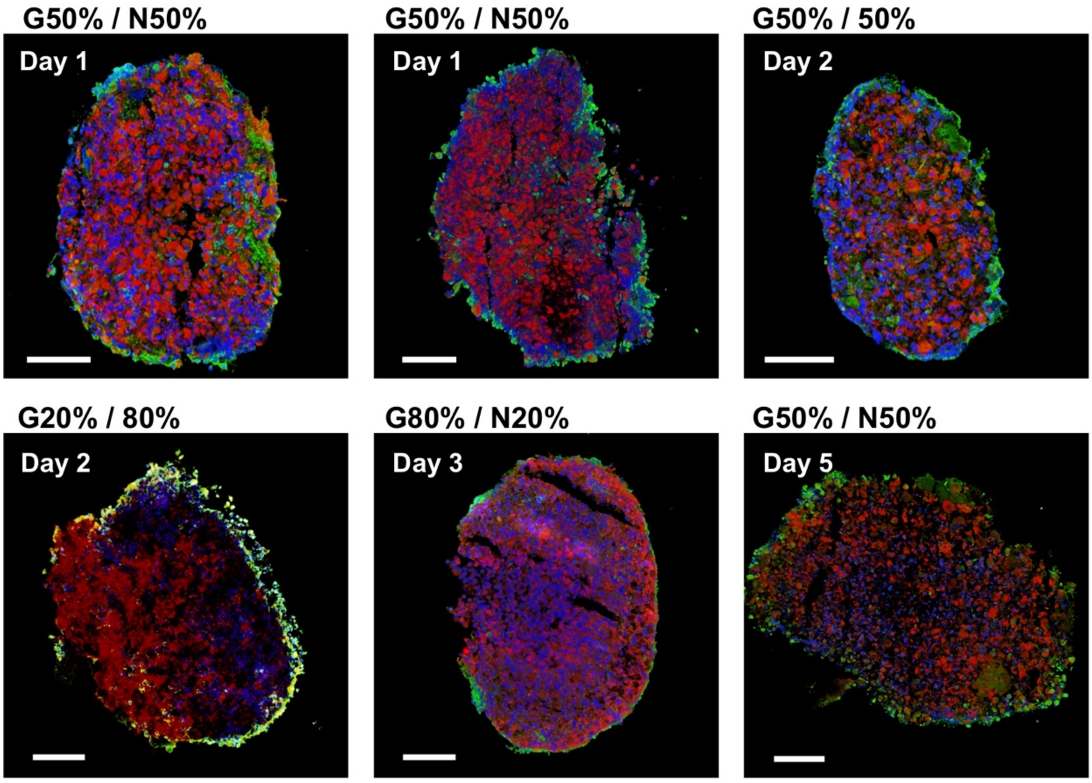

Figure S8. Fluorescence imaging of cross-sections of spheroids of neuronal and glial cells at different intermixing ratios. Staining shows β-tubulin III in green, GFAP in red and DAPI in blue. The green β-tubulin III signal appears more prominently at the spheroid periphery. Scale bars = 100 μm.

A **G100%, day 1**

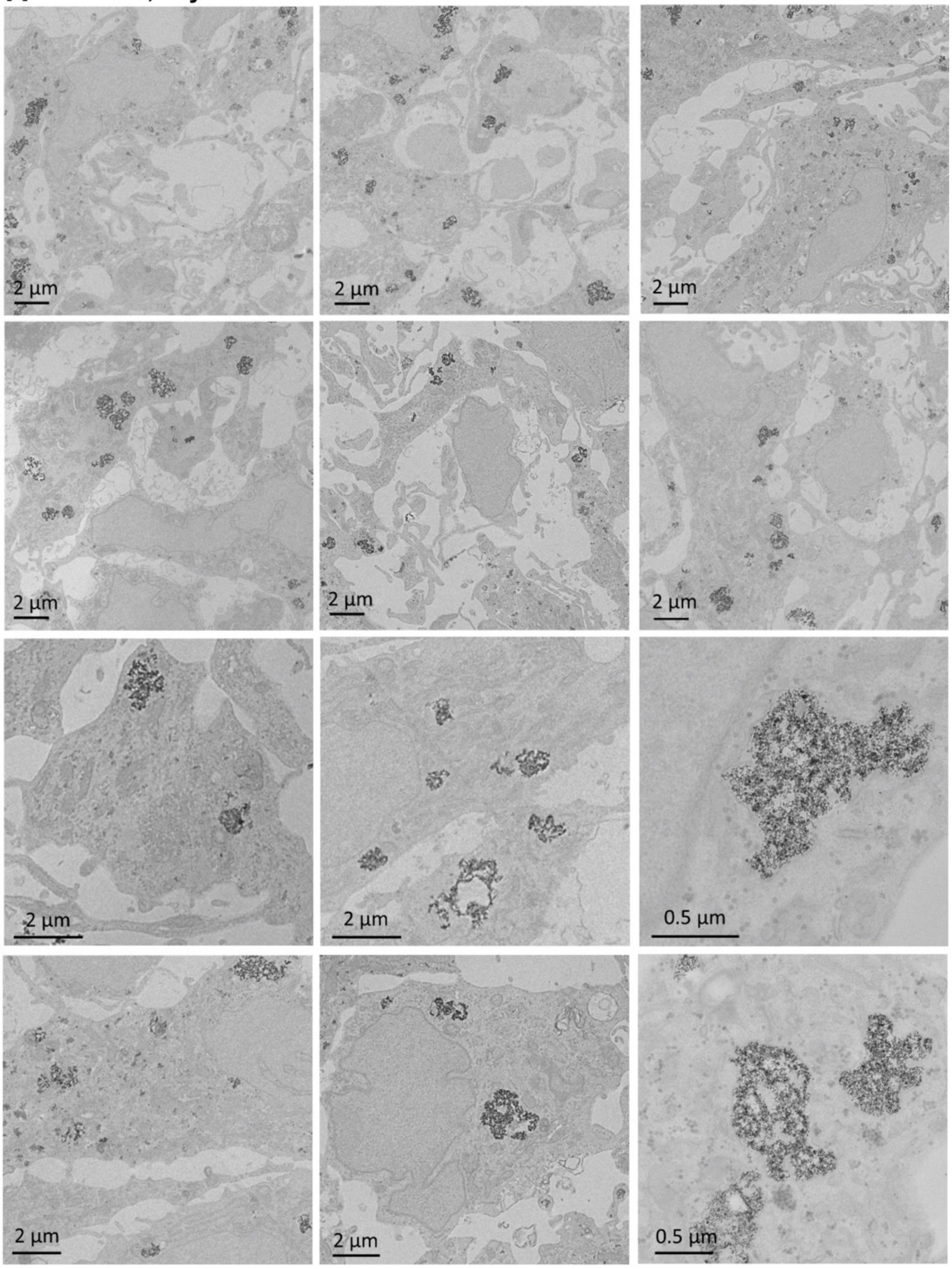

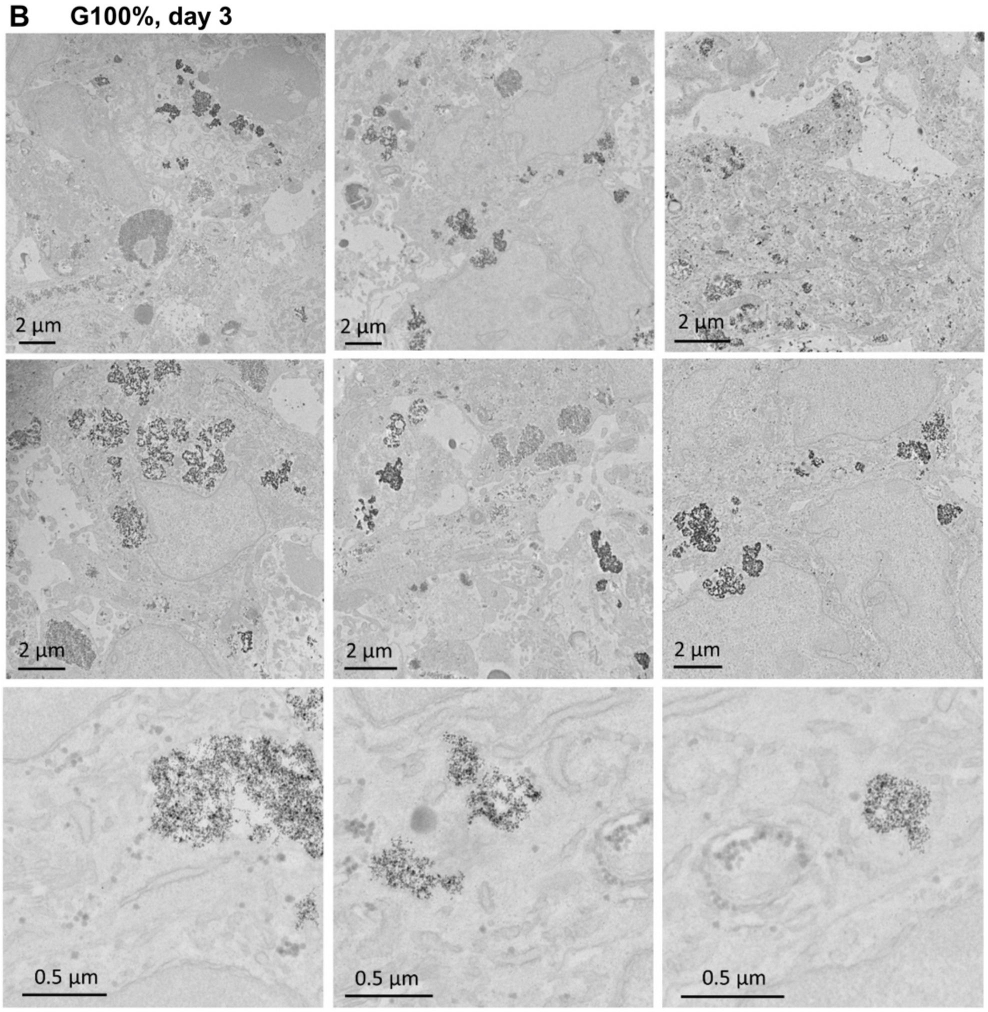

Figure S9. TEM images showing a predominantly endosomal compartmentalization of the nanoparticles in G100% spheroids at (A) day 1 and (B) day 3 of maturation.

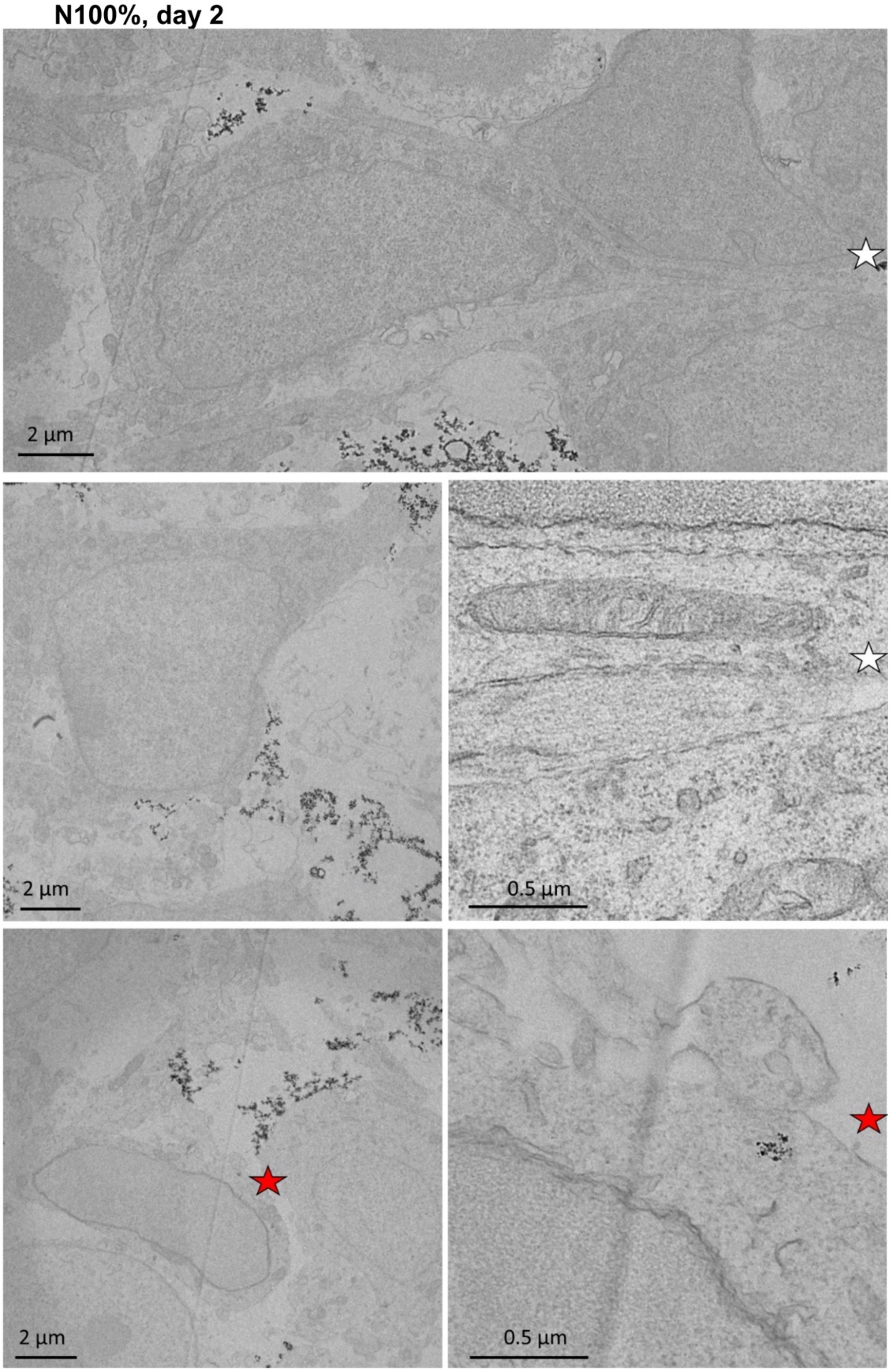

Figure S10. TEM images showing the localization of nanoparticles near the cell membrane in N100% spheroids at day 2 of maturation. Stars indicate concomitant magnification areas.

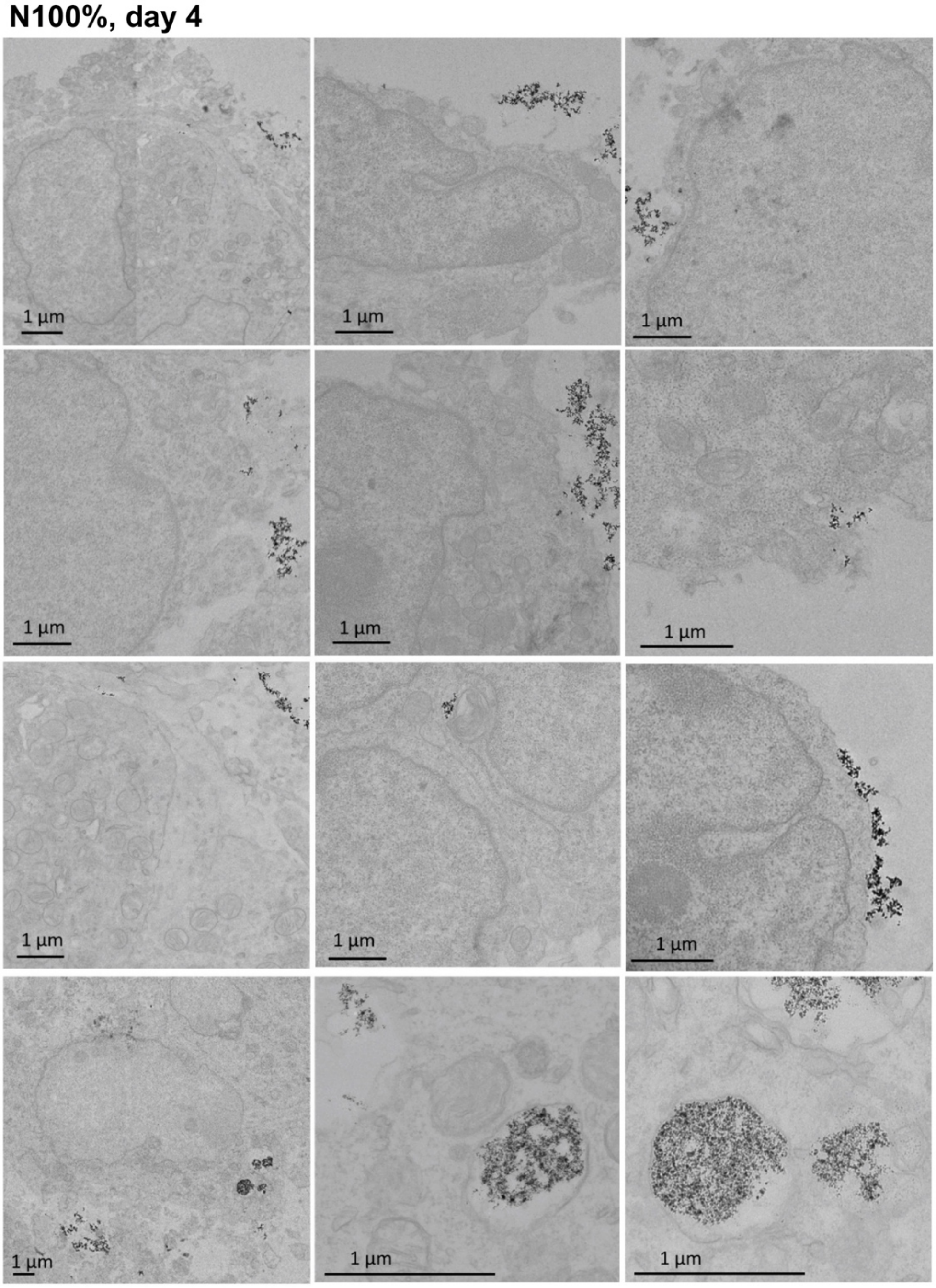

Figure S11. TEM images showing the localization of nanoparticles near the cell membrane in N100% spheroids at day 4 of maturation, and within endosomes in rare instances (bottom panels).

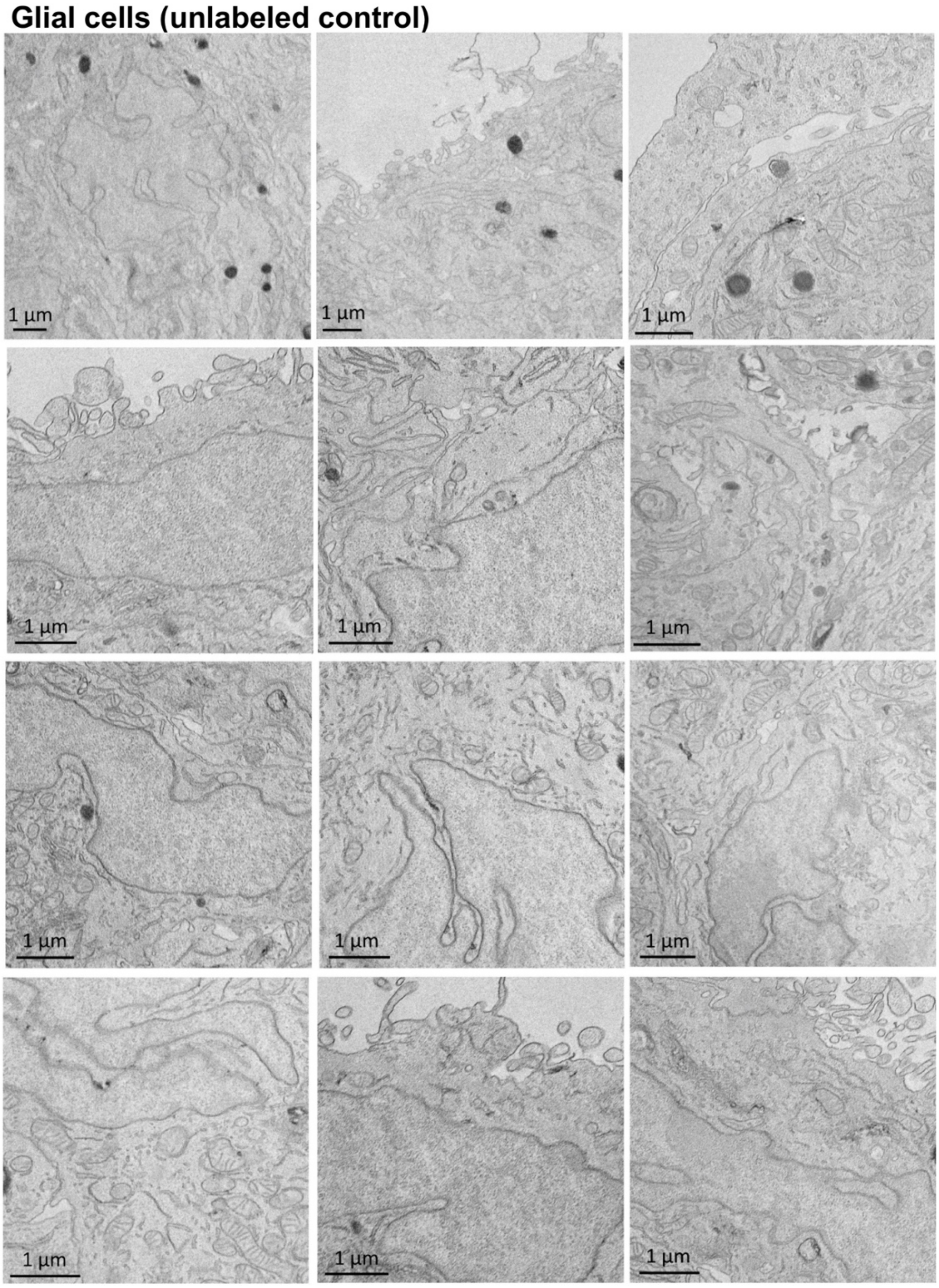

Figure S12. TEM images showing unlabeled (control) glial cells.

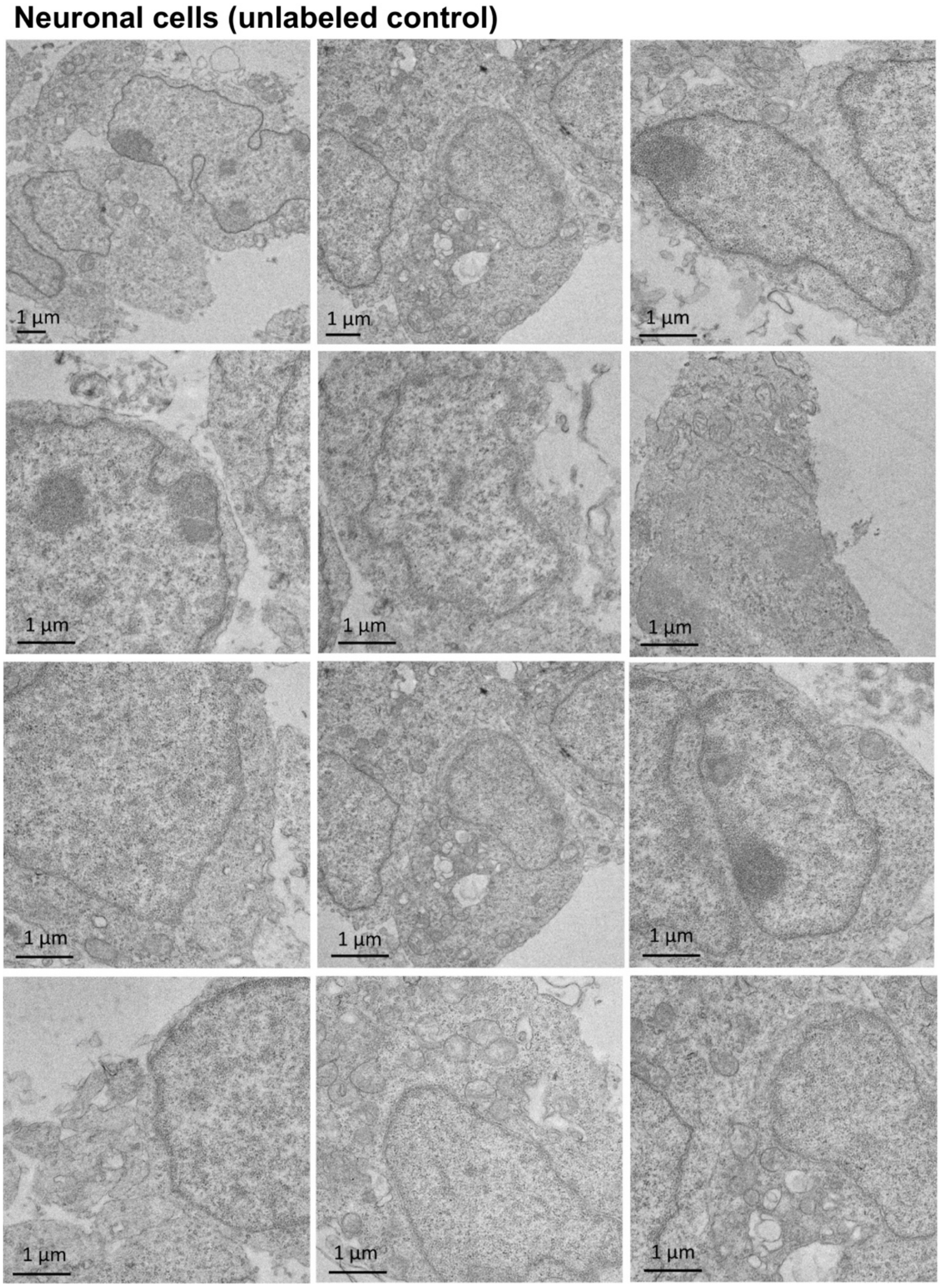

Figure S13. TEM images showing unlabeled (control) neuronal cells.

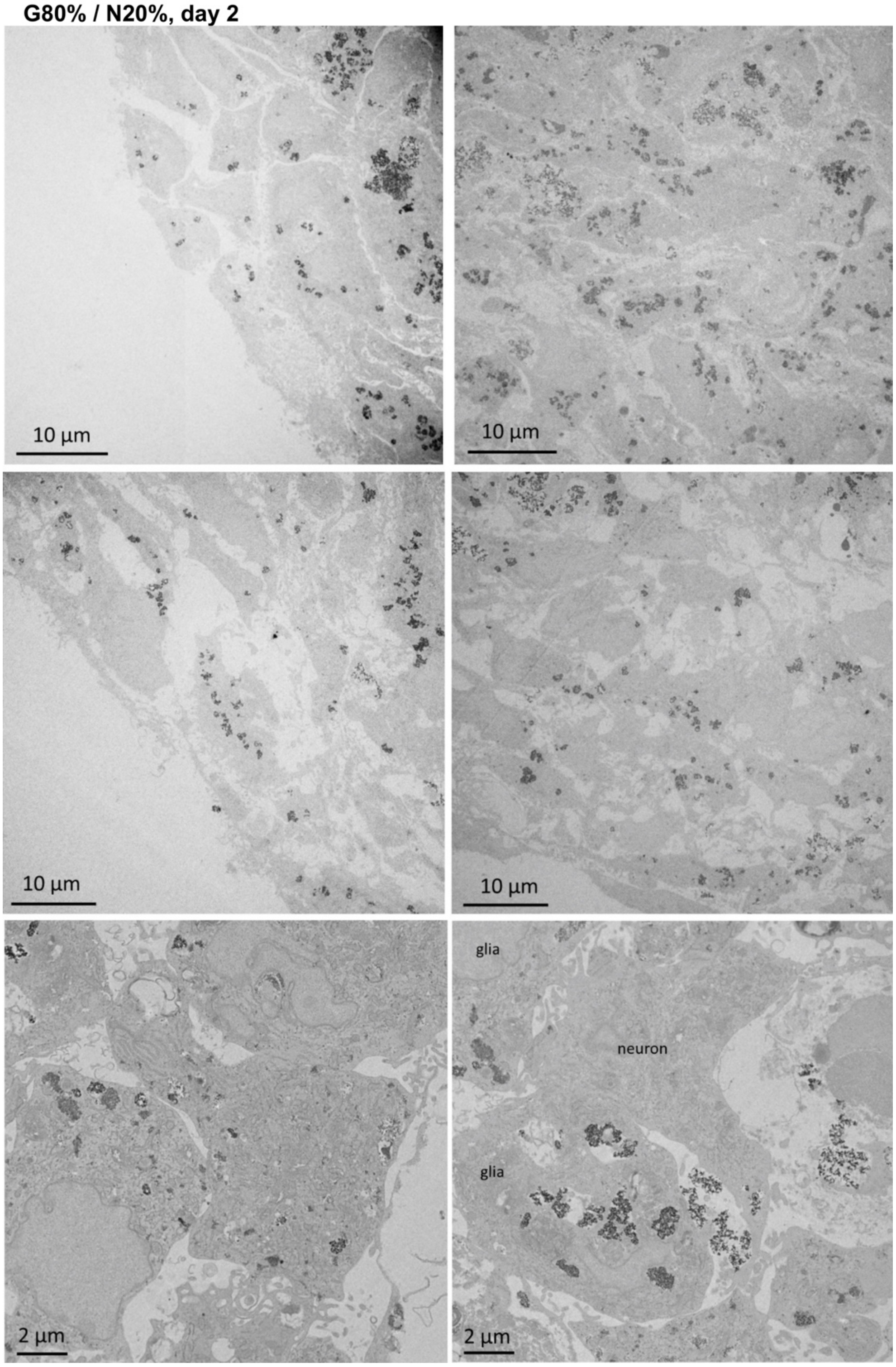

Figure S14. TEM images showing the confinement of nanoparticles within endosomes in spheroids formed after intermixing cells at a ratio of G80% / N20%. The glial cell population can be identified by the presence of magnetic endosomes deep within the cellular structure. In the bottom right panel, a single neuron can be identified according to the nanoparticle localization near the cell membrane (Figure S10).

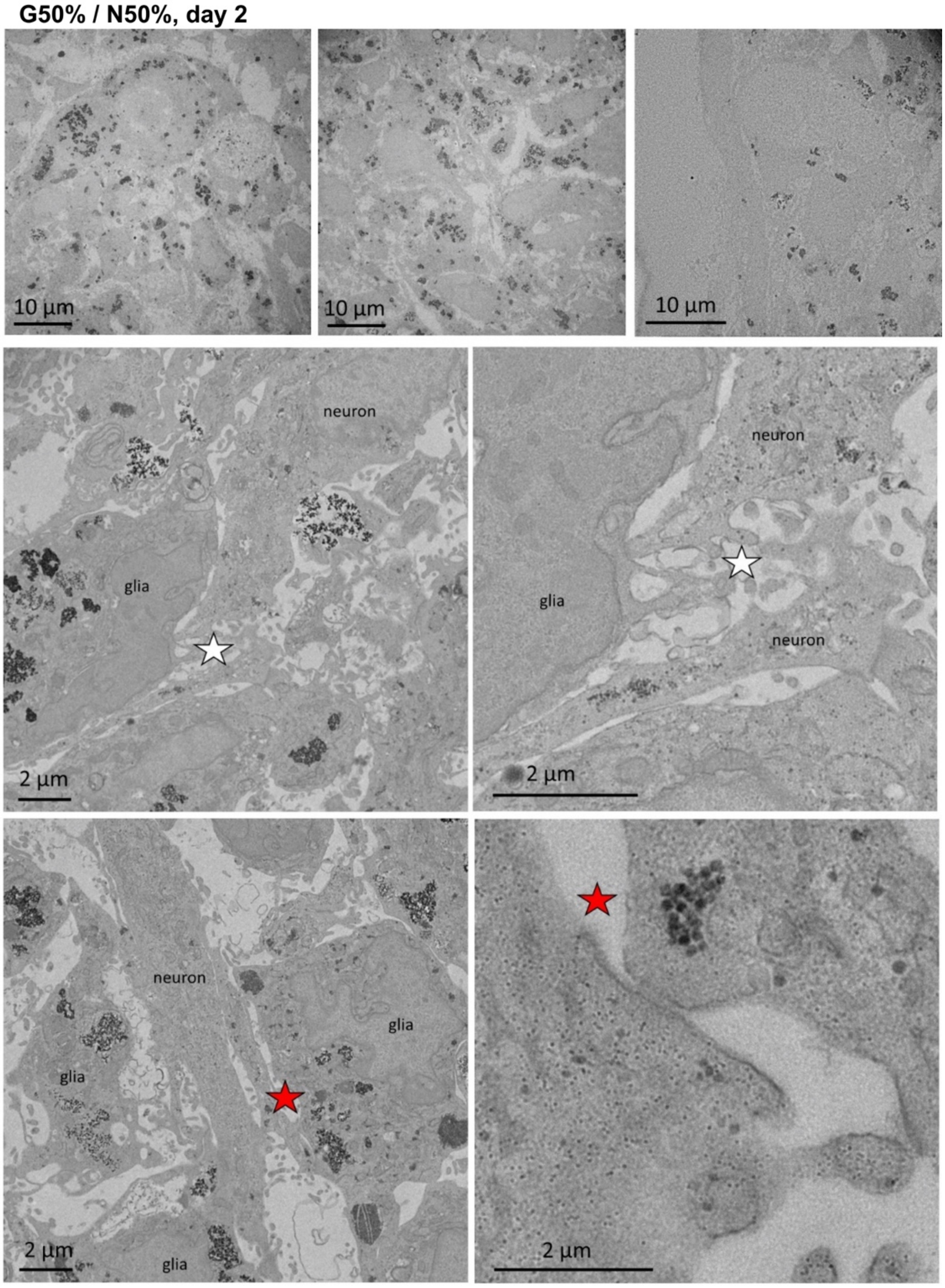

Figure S15. TEM images showing the localization of nanoparticles in spheroids formed after intermixing cells at a ratio of G50% / N50%. Neuron denotation is interpreted based on the localization of nanoparticles, if any. Stars indicate concomitant magnification areas. Neuronal structures can be seen in contact with glial cells in the magnified sections.

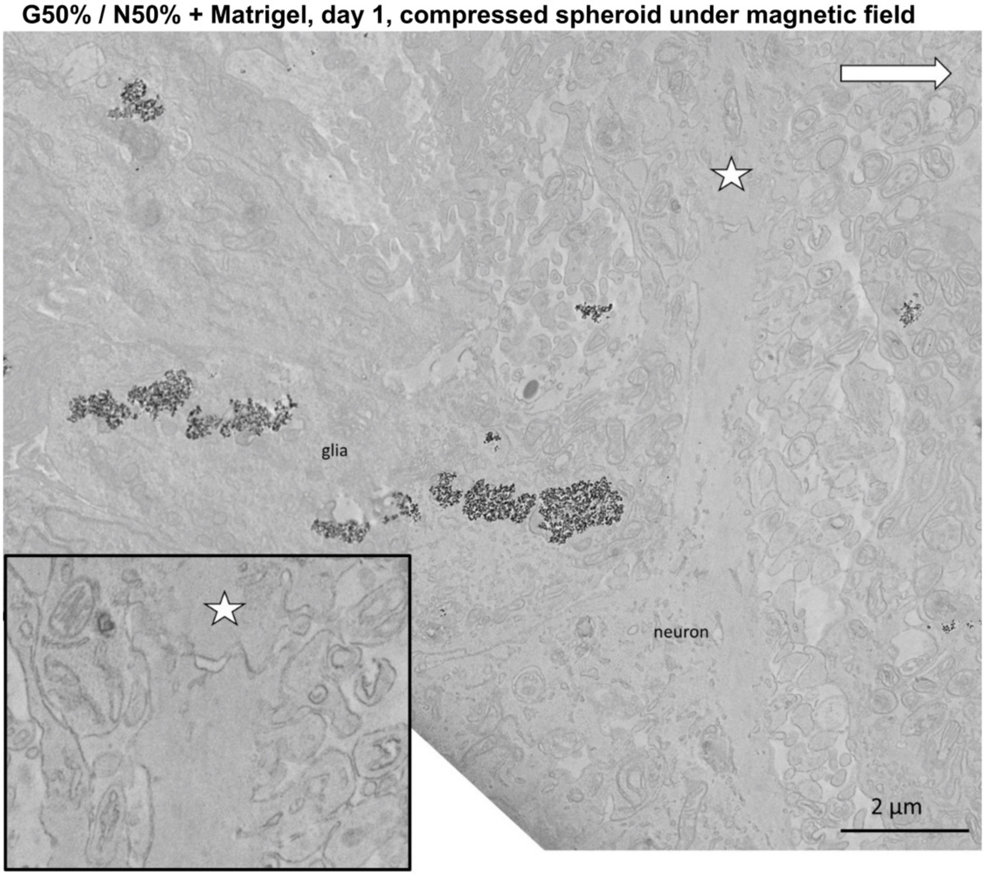

Figure S16. TEM image showing what could be a synapse located at the end of a neuronal axonal body. The cross-section of the spheroid was obtained while in a compressed state after application of the magnetic field gradient (direction of the gradient is indicated by a white arrow). Stars indicate concomitant magnification areas.

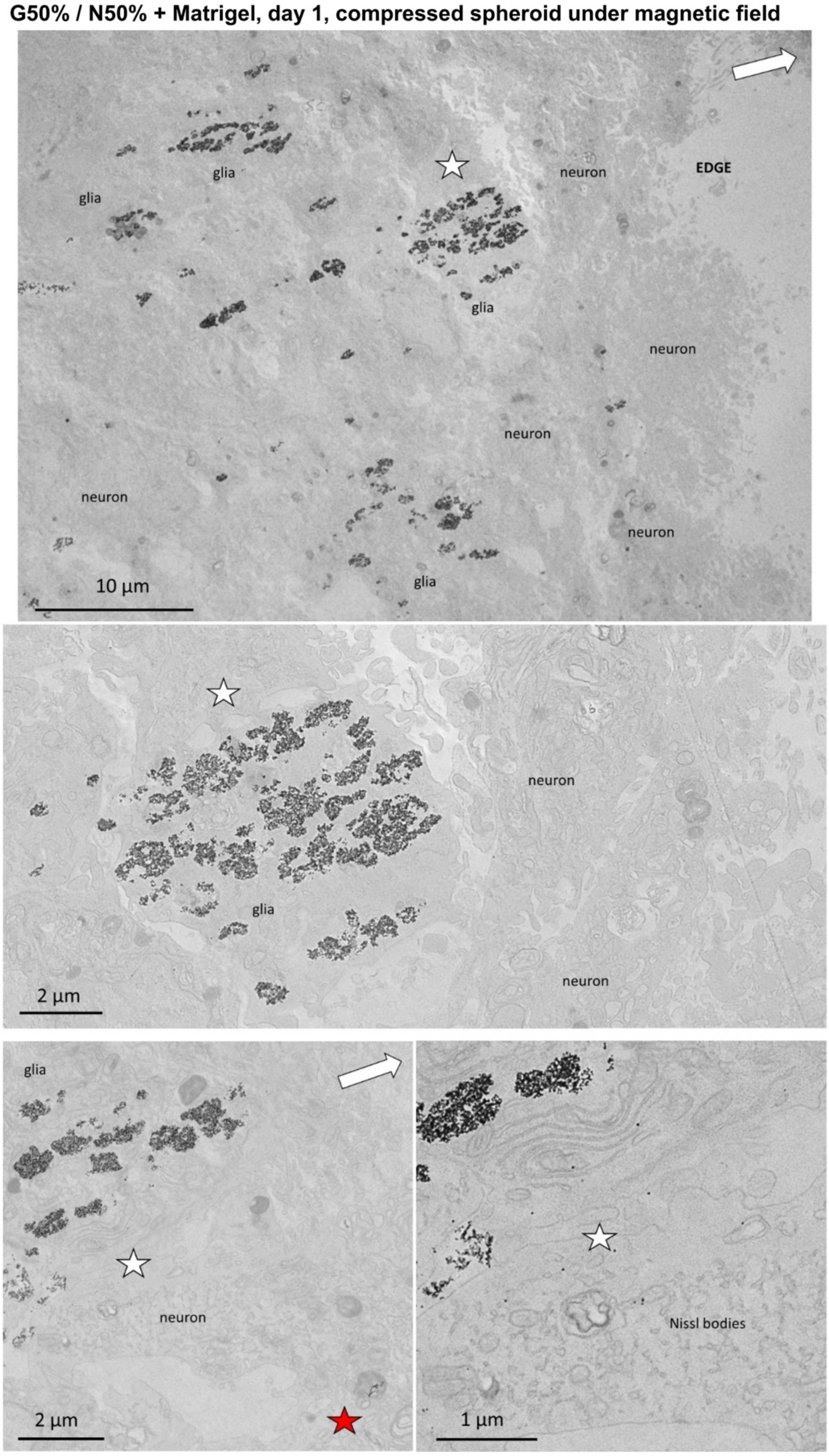

Figure S17. TEM images showing the spheroid cross-section localization of the glial and neuronal cell populations after intermixing at a ratio of G50% / N50% with Matrigel matrix. Cross-section of the spheroid was obtained while in a compressed state after application of the magnetic field gradient (direction of the gradient is indicated by a white arrow). Magnetic endosomes can be observed in alignment with the direction of the magnetic field gradient. Neuronal cells locate mostly at the edges of the tissue section. Nissl bodies can be observed in a neuronal cell (lower panel). Stars indicate concomitant magnification areas.

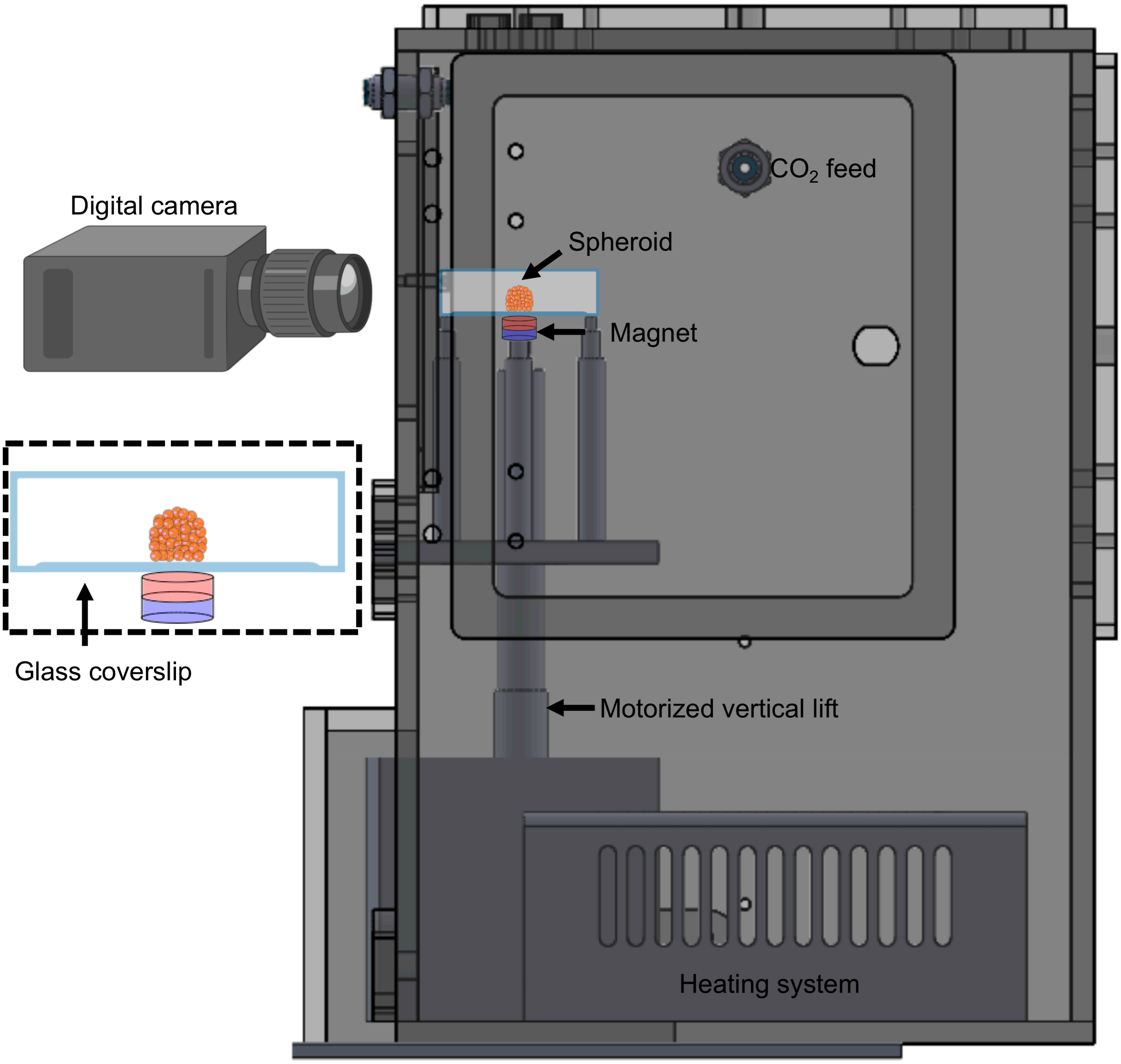

Figure S18. Thermoregulated chamber system used for spheroid compression. The custom-built chamber (Microscope Heaters - Digital Pixel Limited, Brighton, United Kingdom) provides a temperature regulation heating system and a feed for CO_2. A X-VSR20A vertical lift stage (Zaber Technologies, Inc.) was mounted inside the chamber in order to mechanically tune and control the approach of the magnet below the experimentation chamber (direct external contact with the glass coverslip). A Canon EOS R6 digital camera coupled with a Canon MP-E 65 mm f/2.8 1-5x Macro lens was used to image the spheroid compression.

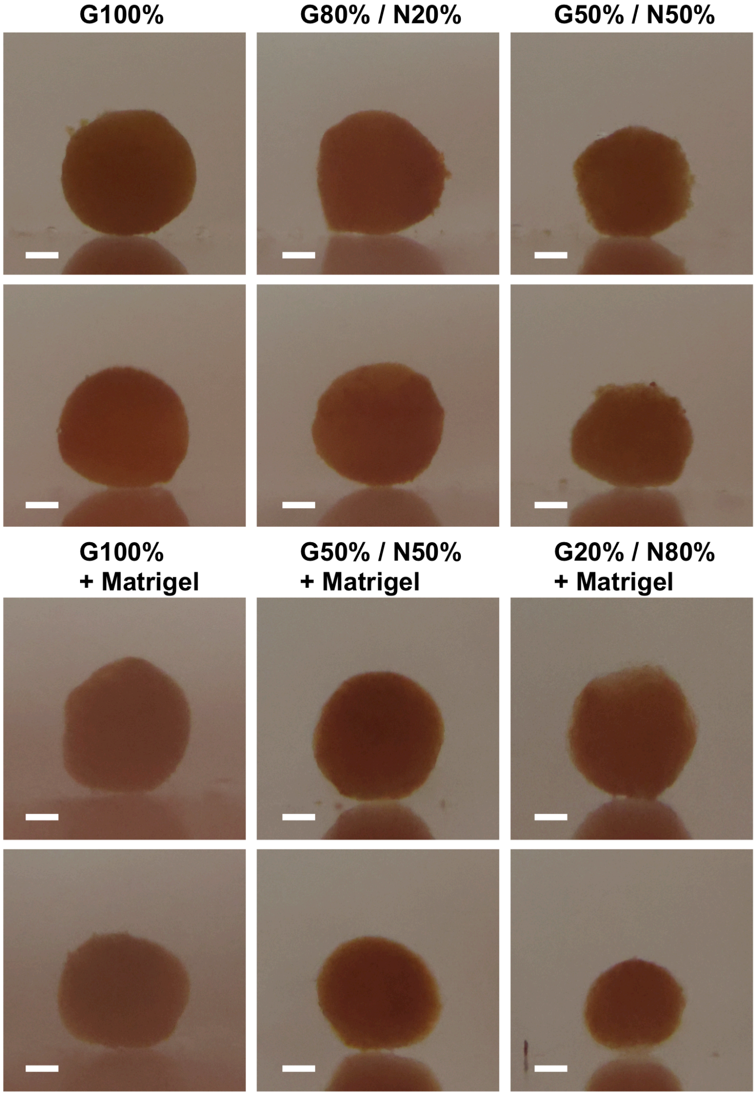

Figure S19. Spheroid lateral control images of different glial and neuronal intermixing ratio spheroids at day 1 of maturation, before application of the magnetic gradient. Scale bars = 200 µm.

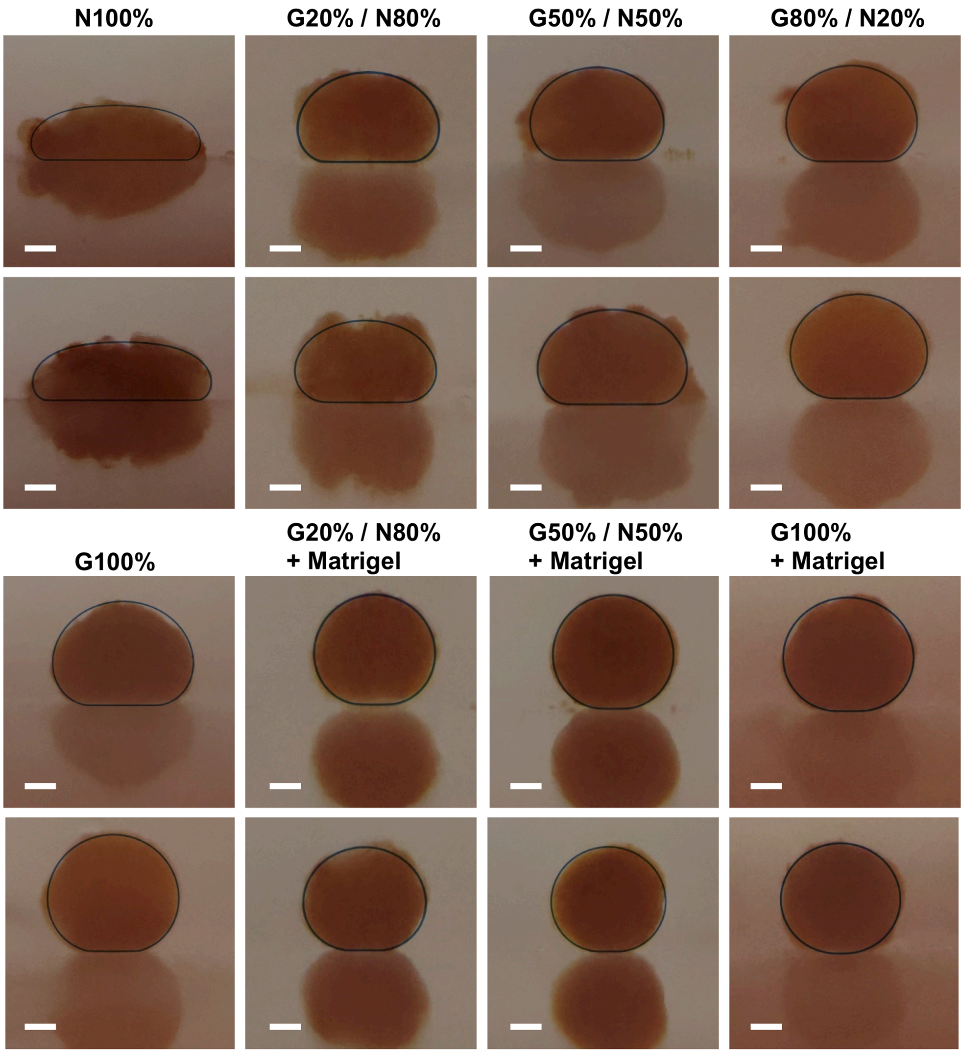

Figure S20. Spheroid lateral compression profiles of different glial and neuronal intermixing ratio spheroids at day 1 of maturation. The profiles were obtained after 5 minutes of magnetic field gradient exposure. Scale bars = 200 µm.
